# Unpacking School-Based Child Sexual Abuse Prevention Programs: A Realist Review

**DOI:** 10.1177/15248380221082153

**Published:** 2022-05-11

**Authors:** Mengyao Lu, Jane Barlow, Franziska Meinck, Lakshmi Neelakantan

**Affiliations:** 1 Department of Social Policy and Intervention, 6396 University of Oxford, Oxford, UK; 2 School of Social and Political Sciences, University of Edinburgh, Edinburgh, UK; 3 Optentia, Faculty of Health Sciences, North-West University, Vanderbijlpark, South Africa; 4 School of Public Health, University of the Witwatersrand, Johannesburg, South Africa; 5 Department of Psychiatry, 6396 University of Oxford, Oxford, UK

**Keywords:** child sexual abuse prevention, school-based interventions, realist review

## Abstract

**Background:** Existing efforts to understand school-based child sexual abuse (CSA) prevention programs mainly focus on the effectiveness of these programs in increasing participants’ CSA knowledge and self-protective skills. There are currently no reviews addressing the underpinning pathways leading to these outcomes. In order to increase our understanding about the underpinning causal and contextual factors and inform the further development of school-based CSA prevention programs, a realist review was conducted to synthesize existing evidence from a broad range of data. **Methods:** An iterative search of electronic databases and grey literature was conducted, supplemented with citation tracking to locate relevant literature. For quantitative evidence, we considered evaluation studies that focused on students aged 5–18 years, who were enrolled in primary or secondary schools; for other types/formats of studies/documents, no population restrictions were applied. We included school-based CSA prevention programs that focused on improving knowledge of CSA or self-protective skills. Outcomes of interest included knowledge of CSA or self-protective skills. We did not apply methodological filters in terms of the types of studies to be included. Thematic content analysis was conducted to synthesize data. **Results:** Sixty-two studies were included. Five themes and five overarching Context-Strategy-Mechanism-Outcome configurations (CSMOs) that contributed to the success of school-based CSA interventions were identified, including tailoring programs to participants’ cognitive developmental levels, repeated exposure of key concepts and skills, utilization of interactive delivery methods and positive feedback, delivery of positive information and application of the ‘train-the-trainer’ model. **Implications:** Findings from this realist review provide insights into the underlying program theory of school-based CSA prevention programs, which can aid in the development and implementation of these programs in the future.

## Background

Child sexual abuse (CSA) is a serious problem for children worldwide. The most recent meta-analysis of 55 studies from 24 countries showed the prevalence of CSA (defined as non-contact abuse, contact abuse, forced intercourse and mixed sexual abuse) to range from 8% to 31% for girls and 3% to 17% for boys (Baker et al., 2013). CSA is associated with a range of adverse psychological, sexual and economic consequences. For example, one meta-analysis found a significant relationship (*d* = .32 to *d* = .67) between CSA and psychological distress (e.g. anger, anxiety and depression) as well as dysfunction (revictimization, self-mutilation, sexual problems, substance abuse and suicidality) in adult women (Manglio, 2009). The recent COVID-19 pandemic has also identified increased risks of CSA, due to social distancing measures, making the identification of effective methods of preventing CSA even more important.

School-based CSA prevention programs are designed to provide participants with the skills to identify, react to and report CSA (David Finkelhor, 2009) and are a widely used strategy to prevent CSA (Walsh et al., 2015). A number of systematic reviews have found that CSA programs are effective in increasing participants’ CSA knowledge and skills regarding self-protection (e.g. Topping & Barron, 2009; Walsh et al., 2015). However, there are now a large number of CSA programs, and little is currently known about the contextual factors or program mechanisms that are associated with better outcomes. This is a significant omission because the effectiveness of school-based CSA prevention programs is highly dependent on the wider contexts within which they are implemented. Schools are ‘complex adaptive systems’ comprised of heterogeneous components including structural (e.g. guidelines, policies and physical environments) and informal elements (e.g. school ethos, social ‘environment’) (Keshavarz et al., 2010). Students’ responses to school-based CSA prevention programs also vary in accordance with their cognitive and behavioural developmental level. Thus, as with medical education interventions, the impact of school-based CSA prevention programs may ‘*vary considerably depending on who delivers it, to which learners, in which circumstances, and with which tools and techniques*’ (Wong et al., 2012, p. 90).

As home to the world’s second largest population of children, China experiences a considerable burden of CSA (Ma, 2018; UNICEF, 2014). While there exists some evidence on the effectiveness of school-based CSA prevention programs in China (e.g. Jin et al., 2017), there is a paucity of evidence regarding the underpinning context and mechanisms that contribute to the intended outcomes. A recent qualitative study (Lu et al., 2020) questioned the effectiveness of such programs given the lack of infrastructure services for children who disclose CSA in China, indicating the need to explore how contextual factors and underlying mechanisms impact intervention efficacy and uptake.

This points to the need to explore underpinning causal factors, in addition to how and in what contexts, school-based CSA prevention programs work, using a realist perspective (Pawson et al., 2005). There are currently no realist reviews of school-based CSA prevention programs. The objective of the current review is to use a realist framework to synthesize existing evidence from both Chinese-language and English-language literature and develop and refine a program theory to explain how contextual factors, intervention strategies and program mechanisms may influence the outcomes of school-based CSA programs. The review question is: what are the contexts, intervention strategies and mechanisms that lead to the success of school-based CSA prevention programs?

## Methods

Realist review, also referred to as realist synthesis, is a theory-driven and interpretive type of literature review (Berg & Nanavati, 2016). It is the secondary research equivalent to realist evaluation (Wong et al., 2012). Realist review has emerged as a popular approach to synthesize evidence on interventions and ‘provides an explanatory analysis of how and why they work (or don’t work) in particular contexts or settings’ (Pawson et al., 2004, p. iv). The realist enquiry posits that, in order to infer a causal outcome (O) between two events (X and Y), one needs to systematically investigate the underlying causal mechanism (M) that connects the events and the contexts in which the events occur (Pawson et al., 2005).

**Figure 1.**
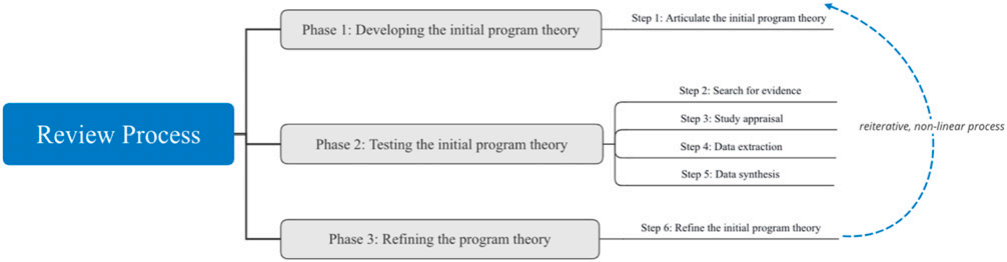
Phases and steps of the realist review.

In this realist review, we expanded the conventional Context-Mechanism-Outcome configuration to include ‘intervention strategies’ (S) as well. Thus, all coded components were aligned using a Context-Strategy-Mechanism-Outcome (CSMO) configuration framework, which was further translated to an ‘*if…then…because*’ statement (Westhorp, 2014). This realist review included three phases and six operational steps which are described below (see Figure 1).

### Phase 1: Clarifying scope and developing an initial program theory

In order to develop an initial program theory, a scoping review of the literature was conducted. The initial scoping phase was intended to serve as a foundation for locating or developing the initial program theory. We examined both included and excluded studies of a systematic review of school-based CSA prevention programs (Lu et al., in preparation), and an earlier systematic review on this topic conducted by Walsh et al. (2015). Having identified eligible studies, we then developed a codebook (available upon request) to guide initial extraction of data to identify relevant factors that could contribute to the initial program theory (Mukumbang et al., 2018, p. 4).

### Phase 2: Search for evidence

#### Search strategy and inclusion criteria

We then systematically searched the following electronic databases from inception to March 30^th^ 2020: EMBASE, Medline, ERIC, EMBASE, the Cochrane Database of Systematic Reviews, PsycINFO, CINAHL, Campbell Database of Systematic Reviews, China Academic Journals Full-text Database and Wanfang Database. We also completed forward and backward citation tracking, contacted authors and searched grey literature such as OpenGrey, abstracts from ProQuest, WHO child maltreatment database, ClinicalTrials.gov and organization websites such as UNICEF and Save the Children. Initial and final search strategies are described in Supplementary Appendix A. We ceased the search when there was sufficient evidence to refine the final program theory.

#### Assessing studies

For inclusion, studies needed to have evaluated school-based CSA intervention programs focused on improving knowledge of CSA, or skill acquisition in self-protective behaviours. Studies had to be published in either English or Chinese. We only included proximal outcomes (CSA knowledge and self-protection skills) in the refined program theory (see Supplementary Appendix F for the examples of data extraction). This was because more distal outcomes, such as decrease of CSA prevalence would require longitudinal data (Gibson & Leitenberg, 2000), and as such, none of the included studies provided such follow-up data. We did not apply methodological filters for study types. For quantitative studies, we considered evaluation studies that focused on students aged 5–18 years, who were enrolled in primary or secondary schools; for other types/formats of studies/documents, no population restrictions were applied because these studies could involve various stakeholders (e.g. teachers, parents and government officials) who might contribute to answering the review questions.

#### Appraisal of primary studies

Studies were selected on the basis of their *relevance* and *rigor*. *Relevance* is defined as whether the study can substantiate and refine the initial program theory. We conceptualized *rigor* as the degree to which included studies were credible based on sample size, data collection and data analysis methods (Pawson, 2006). It should be noted that, however, both *relevance* and *rigor* are not absolute criteria on which a study should be included or excluded. Realist reviews assert that the individual study is rarely the appropriate unit of analysis (Pawson et al., 2005). Therefore, although a study must meet the minimum criteria of *relevance* and *rigor* to be considered for data synthesis, it is suggested that the study as a whole does not get included or excluded on the basis of a single aspect of quality (Pawson et al., 2005).

#### Data extraction

The main characteristics of the included studies were imported into an Excel spreadsheet by the first author. Specifically, data was extracted regarding the descriptions of the programs, the context of the study, intervention strategies used in the programs, underpinning mechanisms of the program, findings of the study and explanations as to how and why the programs worked in particular contexts. The first author conducted data extraction and a second reviewer checked a random subsample of 10% of studies for accuracy. Disagreement between the reviewers was resolved through discussion or in consultation with a third reviewer.

### Phase 3: Synthesis and refinement of initial program theory

Included studies were imported into Atlas.ti (version 9.0.4; Muhr, 2004). We refined the initial program theory as data extraction progressed and iteratively generated how different contexts and intervention strategies appeared to have triggered different mechanisms resulting in different outcomes. We then conducted inductive thematic analysis to identify themes attributed to the program contexts, intervention strategies, mechanisms and outcomes. Thematic analysis is a qualitative data analysis technique, which explores the content of the data and categorizes them into recurrent or common themes (Braun & Clarke, 2006; Green & Thorogood, 2018). We developed each CSMO configuration so that it had supporting evidence in the form of quotes. We also synthesized the key concepts in the included documents into CSMO frameworks through an iterative and open process. We linked these concepts to build a conceptual framework and a final program theory of school-based CSA prevention programs.

We endeavored to bracket our knowledge of CSA prevention and focused on information provided in the included documents. In addition, no document was privileged over others. We conducted constant comparison between the accounts of included documents, to uncover similarities and differences, which subsequently contributed to our refined program theory. We also established an audit trail by keeping reflexive notes throughout the coding process.

## Results

### Initial program theory (Phase 1)

**Figure 2.**
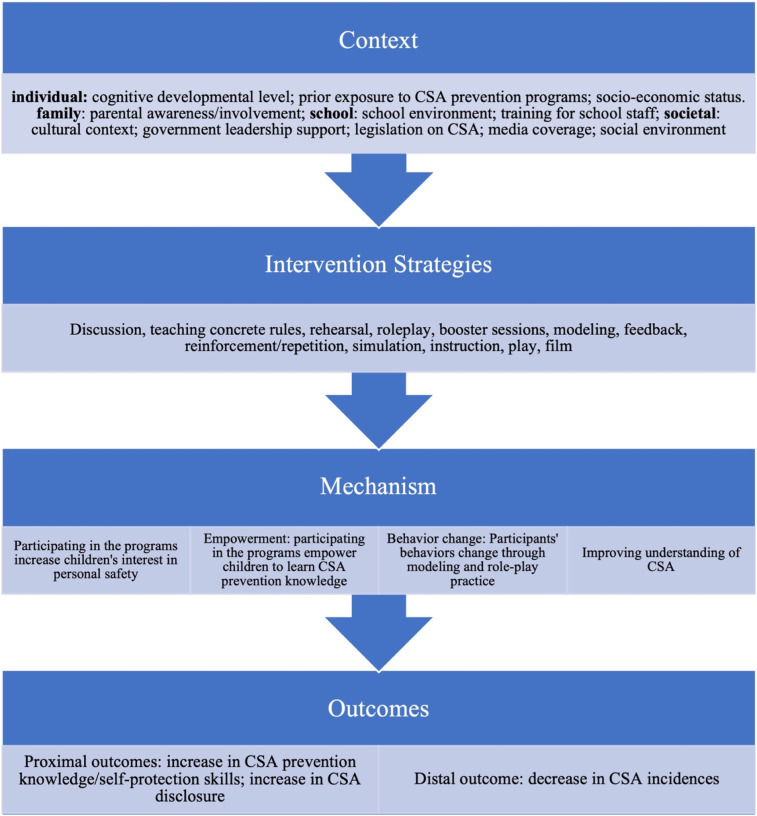
Contexts, strategies, mechanisms and outcomes that emerged from the thematic analysis of the included documents.

A total of 44 studies were included to develop the initial program theory. The characteristics of included studies are listed in Supplementary Appendix C. Figure 2 depicts the key features of the existing literature with regard to the context and program mechanisms and intervention strategic factors, that might influence the outcomes of school-based CSA prevention programs. These concepts were incorporated into our initial program theory, after which we evaluated the extent to which the evidence from the Phase 2 search, confirmed or refuted this theory. In total, we identified four different contextual factors, 12 intervention strategies, four mechanisms, two proximal outcomes (i.e. increase in CSA knowledge/self-protective skills; increase in CSA disclosure) and one distal outcome (i.e. decrease in CSA prevalence). We framed the initial program theory as follows: *If* the participants receive programs that consider their individual characteristics (e.g. cognitive developmental level, prior exposure to CSA prevention concepts and socio-economic status), presence of a supportive family (e.g. parental involvement) and social environment (e.g. cultural contexts where CSA is not a taboo, governmental and legislation support for CSA (C) and key strategies (e.g. discussion, role play and modelling) are applied to the program implementation process (S), *then* participants’ knowledge of CSA or self-protection skills will increase (O), *because* the programs increase their motivation and interest in learning about personal safety, and empower them to learn more about CSA prevention knowledge (M). As a result, their understanding of CSA will change.

### Results of evidence search (Phase 2)

**Figure 3.**
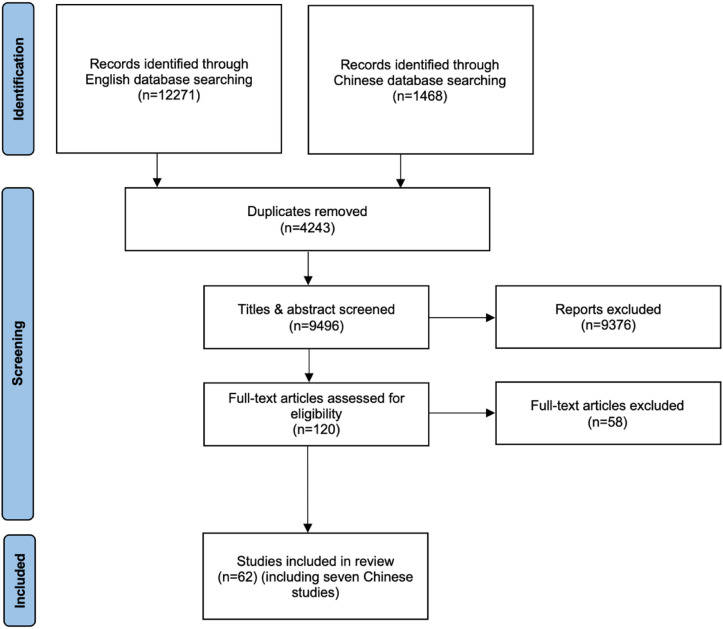
PRISMA flowchart illustrating the search process.

Figure 3 presents the search process of the review. A total of 13,739 studies were found and 9,496 studies were screened after removal of duplicates. One hundred and twenty studies were eligible for full-text assessment. All potentially eligible studies were appraised for relevance and rigor. As a result, 58 studies were excluded due to the lack of conceptual-richness and methodological credibility. In total, 62 studies were included and coded to refine the initial program theory. The summary of the critical appraisal of the included studies is presented in Supplementary Appendix B.

Included studies were published between 1987 and 2020. The included English-language studies were mainly published in the United States, with a small number of studies published in Australia, Canada, China, Ecuador, Germany, Indonesia, the Netherlands, New Zealand, South Korea and the United Kingdom. Among the included studies, there were seven Chinese-language studies. A wide range of documents were included, 57 of which were journal articles, including 38 quantitative studies (e.g. RCTs, pre- and post-test designs), three qualitative studies (e.g. interviews), 15 reviews (e.g. systematic review and meta-analysis, narrative reviews) and one mixed methods study. In addition, we included two commentaries, one critique, one magazine article and one book chapter. Details of the characteristics of the included documents are presented in Supplementary Appendix D.

#### Refined program theory (Phase 3)

Before discussing the CSMO configurations, we first present two overarching concepts that emerged from the thematic content analysis of the included studies.

##### Empowerment

The concept of *empowerment* was frequently used in the included documents as the underlying theoretical basis guiding the development and implementation of school-based CSA prevention programs. Empowerment is a process by which individuals and groups gain greater control of issues of concern to them (Rappaport, 1987). Empowerment encompasses both processes and outcomes and is a multidimensional construct in which each level of analysis depends on the others (Zimmerman, 1995). Included programs demonstrated the empowerment processes by focusing on helping children gain control over their body and safety and enabling them to access supportive resources. Furthermore, included studies discussed empowerment outcomes such as participants’ self-protection skills and behaviors in CSA prevention. For the purpose of CSA prevention, empowerment may be achieved through the following pathways: a) raising children’s awareness regarding CSA; b) establishing trust and a sense of safety; c) providing supporting services and enabling children to develop a sense of control; d) developing children’s confidence and improving self-esteem; e) providing choices and services to enable children to become active agents of change (Simantiri, 2018). Calhoun (2009) examined the relationship between concepts and skills presented in CSA prevention programs. They found that ‘*when given adequate information, a sense of personal power, and a list of community resources, children were able to assist in their own self-protection*’ (Wurtele & Miller–Perrin, 1992; cited in Calhoun, 2009, p. 7). In addition, empowerment was frequently used to conceptualize the objectives of the study. For example:

*‘The goal of the curriculum is to educate and empower students to prevent, recognize, and respond appropriately to bullying, cyberbullying, the four types of child abuse, and digital dangers’* (Bright et al., 2020, p. 5).

Furthermore, *empowerment* appeared to be embedded within key concepts and intervention strategies being used by school-based CSA prevention programs. For example, one of the key messages that prevention programs aim to deliver is that CSA is not the children’s fault, and to *empower* them to share their own experiences, encourage disclosure of negative experiences and enable access to supporting services (Simantiri, 2018). In addition, intervention components such as role play are considered to be at the heart of *empowering* children to prevent CSA because they allow them to apply concepts and transfer them into skills, thus enabling them to stop CSA before it occurs (Kollko et al., 1987).

It should be noted, however, that none of the included studies examined empowerment using a critical understanding of children’s social context, which is considered to be an important aspect of the empowerment process (Zimmerman, 1995). In addition, included studies also failed to explore whether children gain control over their body and utilize self-protection skills in real life CSA situations.

##### Competence

The second concept that emerged from the thematic analysis of the reviewed documents is *competence*. In general, social competence is defined as ‘*involving the personal knowledge and skills which persons develop in order to deal effectively with life’s many choices, challenges, and opportunities*’ (Leffert, Benson, & Roehlkepartan, 1997; cited in Han & Kemple, 2006, p. 241). In early childhood, social competence has been defined as ‘the ability of young children to successfully and appropriately select and carry out their personal goals’ (Guranlnick, 1990, p. 4). Following this definition, the included studies suggest that participation in these programs, enables children to develop personal knowledge and skills to deal with CSA more effectively, thereby enhancing their competence in handling dangerous situations.

The included studies suggest that school-based CSA prevention programs were developed to both *empower* children by enhancing their *competence* in knowledge about CSA and self-protective behaviors (Kim & Kang, 2017).

### Context-Strategy-Mechanism-Outcome configurations (CSMOs)

Once coding was complete, patterns of contexts, mechanisms, strategies and outcomes were identified. The final program theory is presented in Table 1, which has been organized into five commonly occurring themes (i.e. adaptation, repetition, imitation, competence and ownership and trust building) and further refined into five CSMO configurations.

**Table 1.** Final program theory and CSMO configurations.

Theme	Context	Strategy	Mechanism	Outcome	Supporting quotes and data source
Adaptation – CSMO 1	C1: Children’s different cognitive developmental levels	S1: Strategies that tailor to each age group’s needs	M1: Integrating knowledge into cognitive repertoire and learning process	O1: Improvement in CSA knowledge and self-protective skills	‘It is possible that some of the students in the k-3rd group were influenced by other means of accessing knowledge of CSA, which ultimately reduced the effects of the CARE program’ (researcher’s view; Alexander, 1998)
		S2: Strategies that tailor to needs of children with disabilities			
Repetition – CSMO 2	C1: SES	S1: Repeated exposure	M1: Enhancing retention		‘It is vital that teachers and caregivers involve parents in their programs, first by providing them with information about the risks to children and secondly, to encourage the reinforcement of safety concepts in the home’ (researcher’s view; Briggs & Hawkins, 1994b)
	C2: Parental involvement				
Imitation – CSMO 3	C1: Children’s different cognitive developmental levels	S1: Interactive delivery approaches	M1: Engaging in imitation process		‘The success of interventions that performed by nurses in this study was also caused by intervention model which created specifically and compatible with characteristics of primary school aged children, such as love to play, happy to move, enjoy working in groups and happy to feel or do something directly’ (researcher’s view; Neherta et al., 2017)
		S2: Positive feedback			
Trust building – CSMO 4	C1: Supportive environment	S1: Delivery of positive messages	M1: Empowerment	O2: CSA disclosure	‘Desired communication that appeared to lead to higher levels of disclosure included lessons delivered with open body language, warm voice tone, positive behavior management, a capacity to wait for noise to lower naturally, “peer to peer” talk more akin to the playground, short turns taken by presenters and disclosures received without judgment’ (researcher’s view; Topping and Barron, 2003)
Competence and ownership – CSMO 5	C1: Resource-constraint settings	S1: Train-the-trainer model	M1: Competence		‘The “train-the-trainer” approach that our program utilized could allow the trained teacher to continue to reinforce knowledge of CSA prevention strategies for a longer period and with subsequent groups of students beyond the present study’ (researcher’s view; Bustamante et al., 2019)
	C2: LMICS				

*CSMO 1*: Participants’ CSA knowledge and self-protective skills will increase (O) when programs take children’s different cognitive developmental levels (C) into consideration. It is also important to tailor the program to children’s various needs, including 1) special needs for children with disabilities and 2) different age groups (S) such that participants are able to integrate CSA concepts and self-protective skills into their cognitive repertoire and learning processes (M).

*Evidence*: Ten included studies supported this CSMO and Piaget’s cognitive development theory was frequently referenced as being core to the success of school-based CSA prevention programs (e.g. Barbee, 1992; Manheim et al., 2019). This theory suggests that children view and understand the world differently at different periods in their lives and children assimilate the same information in a different manner during different stages of development (Wadsworth, 1996). Included studies that measured skills acquisition and knowledge gains (e.g. Tutty, 2020; Neherta et al., 2017) concluded that programs that used strategies to tailor the content to participants’ cognitive developmental levels and age were more effective in improving participants’ overall performance.

First, for example, an included study that examined the effectiveness of the CSA prevention program *CARE*, found that participants without special needs appeared to gain more knowledge regarding CSA compared with participants with special needs (i.e. remedial students who received support for Language arts) (Alexander, 1998). In addition, authors reported that remedial students tended to overgeneralize prevention messages, which led to emotional stress. It is also suggested that remedial students or students with disabilities would be less able to determine whether they were abused, and to disclose CSA (Martinello, 2014), and that as a result, program content needs to address the needs of these groups of children in terms of increasing their competency in these areas (Martinello, 2014).

Second, age was also identified as an important factor that contributes to better knowledge outcomes. For example, one study which reviewed CSA prevention programs noted that for complex concepts, older students obtained higher knowledge scores than younger students on questionnaires (Kolko, 1988). Instead of abstract concepts, concrete and clear rules with rehearsal (e.g. teaching children to run away when someone asks or touches their private parts) seemed to improve primary school children’s understanding of CSA (Zhang & Deng, 2019). Another suggestion was that older children might already have previous knowledge of the concepts or skills that the program delivers. For example, Alexander (1998) evaluated a school-based CSA prevention program C.A.R.E and argued that it was possible that older children had already accessed knowledge about CSA from other sources, which ultimately reduced the program effectiveness. In a similar vein, using a narrative review that examined the efficacy of school-based CSA prevention programs between 1990 and 2002, Barron & Topping (2008) found that the small outcome gains observed could be attributed to high prior levels of knowledge that participants brought to the program.

*CSMO 2*: Participants’ knowledge of CSA and self-protection skills will increase (O) when the program involves *repeated exposure* to the key concepts (S), which will enhance participants’ understanding of program materials and reinforce key concepts and skills taught in the program (M). The contextual factors (C) that appears to influence this configuration are parental involvement and socio-economic status (SES).

*Evidence*: We identified 18 studies that provided support for this CSMO. Included studies showed that *repetition* and *reinforcement* were necessary to optimize program effectiveness and improve participants’ knowledge outcomes. For example, one study found that there was a need to deliver the program ‘*in a powerful manner*’ (Tutty, 2000, p. 296), including delivering program materials repeatedly or at regular intervals, especially for younger children who were often forgetful of what they had learnt (Manheim et al., 2019; Tutty, 2000; Zhang & Deng, 2019). Repetition of difficult-to-learn concepts was also encouraged in one included study (Barron & Topping, 2008I. Barron & Topping, 2008). However, the evidence also suggests that repetition does not necessarily indicate increased time spent in learning (Davis & Gidycz, 2000); rather, it may refer to the importance of dividing prevention programs into shorter sessions that allow children to stay focused for the entire period, and increases the opportunity to repeat program materials, ultimately leading to greater retention of knowledge. Similarly, an evaluation study of a school-based CSA prevention program (*Talking About Touching*) found that students should not attend programs for one brief session only and that one single session may not be appropriate for all ages (Madak & Berg, 1992).

*Parental involvement* was a significant contextual factor that was viewed as enhancing outcome gains. Two pathways through which parents could engage in the education process were identified: a) involving parents during the program implementation process, such as providing training for parents or informing them of what children have been taught at school (e.g. Kolko et al., 1987; Lape-Brinkman, 1999; Manheim et al., 2019; Xie et al., 2020); b) providing home activities where children can practice key concepts that they learnt in the school with parents at home (e.g. Brown, 2017; Casper, 1999). It is argued that providing caregivers the knowledge and tools to discuss sexual abuse encourages parents to gain understanding in CSA prevention, promoting open dialogue at home. As a result, children feel more comfortable and confident in discussing issues related to CSA (Kenny, 2009; Manheim et al., 2019). Moreover, because the young children have a tendency to forget what they have learnt, it has been strongly recommended that parents participate in CSA prevention education in order to support continual reinforcement of the knowledge at home. In particular, an included study reviewed different types of CSA prevention programs and pointed out that parents must be included in the actual training process and receive CSA prevention training themselves in order to optimize the program effectiveness (Manheim et al., 2019).

The mechanism underlying this intervention strategy was described in one included study as follows:

‘*Involving the family in the educational process may help reduce the secrecy surrounding the topic of CSA, and may stimulate parent-child discussions about sexuality in general…Children trained by their parents would also receive repeated exposure to prevention information in their natural environment, thus providing a series of “booster sessions” to enhance generalization*’ (Wurtele, 1992, p. 874).

The supportive environment was also viewed in included studies to create a safe space for children to disclose CSA that has already taken place or if it occurs in the future. This is discussed further in CSMO 4 below.

SES status was found to be another contextual factor contributing to differences in children’s knowledge gains. In particular, children from lower SES communities were identified in two studies as being at greater risk of CSA due to their lower knowledge and skill base, and as experiencing fewer gains from CSA prevention programs (e.g. Briggs & Hawkins, 1994b; Scholes et al., 2012). Interestingly, we also found that participants’ SES is an important contextual factor influencing parental involvement levels, with less parental involvement identified for students from lower SES families, which further inhibits children’s knowledge gains (e.g. Briggs & Hawkins, 1994a; Sanderson, 2004).

*CSMO 3*: When the program utilizes interactive delivery approaches and positive feedback (S), children will obtain more knowledge of CSA and self-protective skills (O) through imitation (M). The contextual factor concerning this configuration is participants’ cognitive developmental level (C).

*Evidence*: Interactive delivery approaches were identified in 18 included studies as being important factors influencing whether the intended outcomes were achieved. One evaluation study of a CSA prevention program conducted in Indonesia, for example, concluded that the success of the intervention depended on the extent to which the intervention model was designed with the characteristics of primary school aged children in mind (Neherta et al., 2017). Digital program formats that involved interactive learning strategies were also identified as improving participants’ CSA knowledge and self-protective skills. Findings from a study that examined a mobile application designed for preventing CSA among primary school students in South Korea indicated that program materials that were presented using animations and interactive quiz sessions were better received by participants than print-based materials (Moon, Park & Song, 2017). Findings from two Chinese studies found that a program that involved brain storming, role-playing and skills practices (Chen et al., 2013) and games as part of the delivery format (Zhang & Deng, 2019) appeared to increase participants’ knowledge of CSA and self-protective behaviors.

Bandura’s modelling theory was referenced as the theoretical basis for the use of these approaches in a number of papers (e.g. Blumberg et al., 1991; Wurtele, 1987). Bandura & Walters (1977) suggested that programs implemented using actual performance are more likely to be impactful than those are based on symbolic forms of instruction (Wurtele et al., 1986). Bandura also contends that observational learning is more effective if the program content is meaningful to the observer, and they retain the information that makes most sense to them and discard the rest (Barbee, 1992). As a result, when programs use observational learning such as audio or visual materials, older children will be more likely to already have an existing knowledge scheme for incorporating the new knowledge of CSA prevention. Younger children, however, are less likely to make significant behavioral change or knowledge gains based on their limited level of pre-existing knowledge of CSA prevention (Barbee, 1992). Included studies also suggested that prevention programs should not simply use program materials that will be understood by older children (Tutty, 1997); rather, that program educators should make sure that program material is delivered in an engaging and varied manner for young children (Daro, 1994). For example, through involving interactive delivery methods such as modelling, role playing and rehearsal, all of which enable CSA prevention knowledge to be reconstructed and to become a more integrated part of the child’s cognitive organization. These strategies enable children to *engage in imitation* and get children actively involved in the program such that the key information becomes ‘*clearer and more salient, allowing for the most gains in acquisition*’ (Davis & Gidycz, 2000, cited in Manheim et al., 2019, p. 748). It is also suggested that rehearsal provides participants with opportunities ‘*to establish an appropriate sequence of activity while attempting to isolate and discard mistakes*’ (Snyder, 1986, p. 8).

*CSMO 4*: When the environment is supportive (C) and the program consistently delivers messages, such as experiencing CSA is not children’s fault, identifying trusted adults that children could disclose CSA to, and that it’s never too late to disclose (S), participants tend to disclose past or ongoing CSA (O) because they become empowered and therefore are more willing to talk about negative experiences (M).

*Evidence*: Seven included studies supported this CSMO. Disclosure was found to be the most unambiguous outcome of CSA prevention programs (Finkelhor & Strapko, 1992, cited in Alexander, 1998) because when children are well supported, disclosures may stop ongoing abuse and lessen the burden of CSA (Barron & Topping, 2010I. G. Barron & Topping, 2010; Rudolph & Zimmer-Gembeck, 2018). By participating in these programs, children’s knowledge and awareness of CSA are improved (Chen et al., 2012); therefore, they are *empowered* to understand that CSA is not their fault and are more likely to disclose CSA to trusted adults (e.g. Blakely et al., 2019; Kenny, 2009). In particular, one evaluation study conducted in China (Chen et al., 2012) showed that compared with children in the control group, participants who received the CSA prevention program reported that they would tell adults when CSA occurred.

Barron & Topping (2013) who evaluated the effectiveness of a school-based CSA prevention program *Tweenees* stressed the importance of delivering lessons ‘*with open body language, warm voice tone, positive behavior management, a capacity to wait for noise to lower naturally, “peer to peer” talk more akin to the playground, short turns taken by presenters, and disclosures received without judgment*’ (Barron & Topping, 2013I. G. Barron & Topping, 2013, p. 945). A supportive environment also appeared to be an important contextual factor in which disclosure was likely to occur. For example, Blakely et al. (2019) suggested that, at the familial level, children with supportive caregivers were more likely to disclose CSA. Barron & Topping (2013) proposed that at the program level, incorporating proactive statements within a supportive environment where ‘*students hear others disclosing and presenters respond with affirming communication*’ (Barron & Topping, 2013I. G. Barron & Topping, 2013, p. 944) would likely lead to disclosure.

*CSMO 5*: When the ‘train-the-trainer’ model is integrated into the program implementation process (S), participants’ knowledge and skill gains increase (O) as it enhances trainers’ competence and ownership of the program (M), thus allowing for further reinforcement of concepts beyond the study. The contexts (C) under which this CSMO is most effective include but is not limited to settings where resources are constrained (e.g. lower- and middle-income countries (LMICs)).

*Evidence*: The ‘train-the-trainer’ model refers to experts in a particular field instructing others in the community, school or other settings on how to implement a prevention program (Baker et al., 2014). As discussed in CSMO 2, parents and caregivers can be empowered through an increase in their knowledge and skills. Their involvement can be viewed as an example of the application of the ‘train-the-trainer’ model. In this section, we primarily focus on school settings where teachers are the ‘trainers’ of CSA prevention programs. We found 15 studies supported this CSMO.

In the included studies, teachers were identified as being critical in the prevention process because children are required to attend school and teachers have the closest contact with children outside of their homes (Scholes et al., 2012). Integration of the ‘train-the-trainer’ model into the program implementation process was also found to enable program deliverers to be trained to teach the program materials following the protocol, which in turn was identified as increasing teachers’ CSA knowledge and chances of having a successful and rewarding experience, and thereby also of having an impact on how the program materials are delivered to students (Baker et al., 2013). Involving teachers in the program implementation process and providing them with training was identified as improving the sense of teachers’ *ownership* of the program, and thereby of deepening their understanding of the program materials, thus allowing for further reinforcement of concepts beyond the study (Barron & Topping, 2008I. Barron & Topping, 2008).

Findings from LMICs and resource-constrained settings offer unique evidence for this CSMO configuration. In addition to promoting the long term impact of the program, the ‘train-the-trainer’ model was identified as being an important means of ensuring that these programs are delivered in settings where there is a shortage of educators because ‘*it helps to minimize time and expense required of outside staff to implement the program as well as harnessing the relationships that already exist between teachers and students*’ (Orfaly et al., 2005, cited in Baker et al., 2013, p. 169). This is particularly important in contextual settings where there is a lack of professionals with expertise in CSA prevention and a lack of teachers’ confidence in delivering these types of programs (e.g. Chen et al., 2008; Yang, 2019; Yi et al., 2020). The effectiveness of the ‘train-the-trainer’ model was illustrated by one included study in which a sexual violence prevention program with high school students in Hawaii was delivered by teachers using the 'train-the-trainer' model, and which showed that students’ knowledge of sexual violence in the intervention group significantly improved after the study, indicating that training teachers to become program deliverers is promising in achieving the program’s intended outcomes (Baker et al., 2013). This was also confirmed in a study with elementary school teachers in Beijing which found that when provided with comprehensive training and sufficient support, teachers were able to deliver the program effectively (Jin et al., 2017). For instance, one study that reviewed the current situation in terms of CSA prevention education in primary schools in China, highlighted the need for professionals who can provide training to teachers and school staff, in order to improve teachers’ knowledge in CSA prevention and facilitate the effective delivery of CSA programs (Yi et al., 2020). Furthermore, the effectiveness of the ‘train-the-trainer’ approach was confirmed as part of an evaluation with a group of elementary school teachers in Beijing, which found that when provided with comprehensive training and sufficient support, teachers were able to deliver the program effectively (Jin et al., 2017).

Furthermore, researchers pointed to the usefulness of the model in cultural settings such as Hawaiian and Asian communities, which emphasize the importance of extended family networks, suggesting that the ‘train-the-trainer’ model has the potential to be utilized in settings other than schools (see parental involvement explained in CSMO 2). Similarly, Bustamante et al. (2019), who evaluated the effectiveness of a school-based CSA prevention program that utilizes the ‘train-the-trainer’ model with children aged seven to 12 years old in Ecuador, found that knowledge and skills were maintained at the six-month follow-up, suggesting that the ‘train-the-trainer’ approach allowed for further knowledge/skills reinforcement beyond the study.

*Linkages between CSMOs*: It should be noted that, while we did not examine the interactions across configurations, they appeared interrelated and could be present in one program in any combination. For example, ‘*repeated exposure*’ is proposed as a separate configuration but is also linked to CSMO 5, which discusses the train-the-trainer model. Similarly, CSMO 1 suggests that programs should be tailored to participants’ cognitive developmental levels, which echoes CSMO 3 which highlights the use of interactive delivery methods for different age groups.

## Discussion

### Study Findings

This realist review was the first attempt of which we are aware, to examine how, why and under what circumstances, school-based CSA prevention programs work in order to assess the extent to which they provide children with the knowledge about CSA prevention and self-protective skills. A range of study designs and publication types were included to deepen our understanding regarding the underlying program theory (Pawson et al., 2005). Furthermore, the concepts of ‘relevance’ and ‘rigor’ were used to assess the quality of the studies, which resulted in a total of 62 studies being included for the purpose of developing the final program theory.

The initial program theory was developed in the first stage of the review based on studies included in an earlier review by Walsh et al. (2015) and consisted of a suggested series of CSMOs that were then refined in the second phase. As described in the methods section, we strengthened the CMO configurational logic of the realist synthesis by incorporating ‘intervention strategies’ into the original CMO configuration. This kind of modification has been proposed in previous realist reviews. For example, Velonis et al. (2020) incorporated ‘strategies’ into the CMO configuration to illustrate the process through which batterer interventions work.

We then identified two overarching concepts underlying these intervention programs that emerged from the included studies and documents, namely, ‘empowerment’ and ‘competence’. These were included in the final program theory as core underpinning concepts. Following this, we developed five CSMO trajectories that appear to underlie school-based CSA prevention programs. These highlight the importance of tailoring the program to the cognitive developmental level of the participants, repeated exposure to program material, the use of interactive delivery approaches and positive feedback, delivery of positive information and the utilization of the ‘train-the-trainer’ model.

Our refined program theory not only substantiated the initial program theory, but also provided more nuanced configurations that were supported by the wider literature. First, we argued that participants are able to integrate the taught concepts regarding CSA and self-protective skills into their cognitive repertoire and learning processes when the intervention program is tailored to their cognitive developmental level, which will lead to the improvement of participants’ CSA knowledge and self-protective skills. Second, if participants are repeatedly exposed to CSA prevention knowledge and self-protective skills, the mechanism of knowledge reinforcement and knowledge enhancement can be triggered, thereby promoting achievement of the intended outcomes. We also identified that SES and parental involvement are two major contextual factors in which this configuration is likely to be activated.

Third, we proposed that through the use of interactive delivery methods and positive feedback, participants are able to engage in a process of imitation that enables them to improve their overall performance. The contextual factor in which this configuration was more likely to be triggered included participants’ cognitive developmental levels. Fourth, we argued that when positive information is delivered, children will feel more empowered sharing negative experiences, thereby leading to the disclosure of CSA. A supportive environment was identified as being an important contextual factor for this configuration. Last but not least, we suggested that particularly with LMICs and resource-constrained settings, the use of the ‘train-the-trainer’ model ensures that more skilled providers such as teachers are able to deliver the CSA programs effectively.

### Strengths and Limitations

The main strength of this review was the use of a realist methodology. To the best of our knowledge, this is the first use of a realist review to explore the CSMOs that underpin school-based CSA prevention programs. Unlike conventional systematic reviews, which focus on the effectiveness of the programs, we included a wide range of study designs to deepen our understanding regarding contextual, strategic and mechanistic factors that lead to the success of these programs. By unpacking the program using a realist lens, we have identified some of the key features of CSA prevention programs. The included studies covered a range of diverse contexts and populations. Further, our review did not only include studies published in English, but also made use of studies published in the Chinese literature, highlighting the similarities in CSMOs across contexts.

There are three major limitations of the review worth noting. First, in order to be comprehensive, we searched all databases and other sources from inception, and as a result, some of the programs in this review were unavailable or no longer accessible. Furthermore, it is possible that studies published in languages other than English and Chinese may have been missed. Second, it should be noted that the purpose of this review was not to provide a detailed account of all existing or potential relationships between CSMOs in school-based CSA prevention programs but rather to identify from the existing literature some of the key mechanisms; it was also not aimed at providing evidence of the effectiveness of the CSMOs identified and as such, this review is not conclusive and needs to be complemented with further studies to test and refine the program theories that we have developed here. Third, we generated our initial program theory by examining studies in two recent systematic reviews on this topic (i.e. Walsh et al., 2015; Lu et al., in preparation). We addressed problems related to this approach at the theory refinement stage where we systematically searched for studies with a range of types of research designs. However, it should also be noted that, our refined program theory was based on existing literature and did not search for studies beyond those who had direct relevance. For example, we only searched for child-focused interventions and excluded interventions such as adult interventions which might have included elements relevant to the program theory. However, by triangulating findings from across 61 documents, we believe that the CSMOs developed in this realist review reflected the underlying pathways most relevant to school-based CSA prevention programs. Last, it was not possible to involve a broad range of stakeholders in the review process. Instead, we contacted study authors to request additional information on the intervention implementation process. Furthermore, all members in the review team have expertise in the field of child protection/abuse prevention, which was utilized when developing the initial and final program theory.

### Implications for Future Research, Policy and Practice

First, program developers should utilize the CSMOs derived from this realist review to inform future program development and implementation. CSA programs should be tailored to the cognitive developmental level of the targeted participants, using repeated exposure of key concepts and skills, and utilization of interactive delivery methods and positive feedback, and delivery of positive information. It is also important that policy makers in LMICs commission the delivery of programs that are optimized in terms of the needs of their particular populations, including the use of programs that can be delivered using ‘train the trainer’ models, particularly in settings where there is a shortage of program providers.

Second, it should be noted that the current realist review included two most commonly used proximal outcomes measured by questionnaire-based and vignette-based instruments (i.e. increase in CSA knowledge/skills; increase in CSA disclosure). There are, however, other child outcome measures used when evaluating the effectiveness of school-based CSA prevention programs, including children’s perception of safety, self-efficacy/confidence to act and harm (Trew et al., 2021). Further to this, the widespread school closures in 131 countries due to the COVID-19 pandemic (UNESCO, 2018) has posed additional challenges for implementing and evaluating school-based CSA prevention programs. As such, future studies should consider including the measures highlighted above and measures particularly relevant to the current context in their realist synthesis, which might provide additional insights into the underlying mechanisms of CSA prevention programs including online CSA prevention programs, especially during periods such as a global pandemic. Similarly, this realist review did not consider distal outcomes such as decrease in CSA prevalence due to lack of eligible studies. In other words, our final program theory speaks to the proximal outcomes (i.e. knowledge of CSA prevention and self-protective skills) of school-based CSA prevention programs.

Last but not least, future research should ensure that the reporting of the study process provides a clear picture of the underlying program theory thereby allowing for a deeper understanding of whether the necessary program mechanisms are in place to achieve the desired outcomes. Future investigations should further explore the contextual factors that determine the success of the intervention programs.

For LMICs context, there is a need for more rigorous studies to be conducted in the CSA prevention field, including high-quality qualitative studies that explore stakeholders’ perspectives on school-based CSA prevention programs, evaluation studies that rigorously examine the effectiveness of these programs and systematic reviews that examine the overall effectiveness of these programs. The proposed combination of qualitative and quantitative studies are necessary to develop a comprehensive understanding of the way in which school-based CSA prevention programs work in a LMICs context. Further, our findings suggest that teachers and parents can be effective agents of change to facilitate the delivery of school-based CSA prevention programs, which is important in LMICs and resource-constrained settings. Therefore, future practice in these settings should involve engaging teachers, as program providers. However, it should be noted that perpetrators maybe someone the child knows and/or may live in the household. In fact, previous research has suggested that up to one-third of CSA is perpetrated by family members (Seto et al., 2015). Last but not least, given that the primary out-of-school rate is 20% in low-income countries compared with only 3% in high-income countries (UNESCO, 2018), there is a need to find other ways of preventing CSA that go beyond school settings.

## Conclusion

Although school-based CSA prevention programs have been extensively researched since their development in the 1980s, there is still a lack of evidence regarding the theoretical underpinning in terms of how these interventions work, for whom and in what circumstances. This realist review has enabled us to identify key contextual factors, program strategies and program mechanisms that appear to be associated with improvements in CSA knowledge and self-protective skills in children. The implications for future policy, practice and research are identified.

### Critical findings

• Five CSMO trajectories appear to underlie school-based CSA prevention programs: tailoring programs to participants’ cognitive developmental levels, repeated exposure of key concepts and skills, utilization of interactive delivery methods and positive feedback, delivery of positive information and application of the ‘train-the-trainer’ model.

### Implications

• Program developers should utilize the CSMOs derived from this realist review in future program development and implementation.
• Future research should ensure that the reporting process provides a clear picture of the underlying program theory thereby allowing for a deeper understanding of whether the necessary program mechanisms were in place to achieve the desired outcomes.
• More rigorous studies need to be conducted in the CSA prevention field to develop a comprehensive understanding of the way in which school-based CSA prevention programs work. Practice in LMICs and resource-constrained settings should involve engaging teachers, as program providers.

## Supplemental Material

sj-pdf-1-tva-10.1177_15248380221082153 – Supplemental Material for Unpacking School-Based Child Sexual Abuse Prevention Programs: A Realist Review Click here for additional data file. Supplemental Material, sj-pdf-1-tva-10.1177_15248380221082153 for Unpacking School-Based Child Sexual Abuse Prevention Programs: A Realist Review by Mengyao Lu, Jane Barlow, Franziska Meinck and Lakshmi Neelakantan in Trauma, Violence, & Abuse
