# Supplementary material for: Unpacking School-Based Child Sexual Abuse Prevention Programs: A Realist Review
Source: Trauma Violence Abuse. 2022 May 11;24(4):2067–81. doi: 10.1177/15248380221082153 (PMC10486164; doi:10.1177/15248380221082153)
Supplement: sj-pdf-1-tva-10.1177_15248380221082153 – Supplemental Material for Unpacking School-Based Child Sexual Abuse Prevention Programs: A Realist Review [file sj-pdf-1-tva-10.1177_15248380221082153.pdf]

Appendix

Appendix A. Search terms

Initial search terms in English databases

Search Number	Search
#1	(pupil* OR child* OR adolescen* OR young people OR young person OR youth* OR teen* OR student* OR school aged child* OR schoolchild* OR school children OR boy* OR girl* OR schoolboy* OR schoolgirl* OR kindergarten* OR pre-school* OR pre-school OR school* OR secondary OR primary OR pre-school*).ti/ab
#2	(sexual abuse OR sexual assault* OR sexual maltreat* OR sexual molest* OR sexual offen* OR rape OR sexual coerc* OR forced sex OR sexual harassment OR sexual exploit* OR sexual victim* OR sexual crim* OR groom*).ti/ab
#3	(prevention* OR activit* OR program* OR intervention* OR educat* OR curriculum* OR treatment*).ti/ab
#4	#1 AND #2 AND #3

Initial search terms in Chinese databases

Search Number	Search
#1	(儿童 OR 少年 OR 青少年 OR 学龄* OR 学生 OR 男孩 OR 女孩 OR 幼儿园* OR 小学生 OR 初中生).ti/ab<br>(child* OR adolescent* OR teen* OR pre-school child* OR school aged child* OR boy* OR girl* kindergarten* OR primary school student* OR secondary school student*).ti/ab
#2	(性侵犯 OR 性骚扰 OR 性侵害 OR 性暴力 OR 性虐待 OR 性犯罪).ti/ab (sexual abuse OR sexual harassment OR sexual assault OR sexual violence).ti/ab
#3	(预防 OR 干预 OR 活动 OR 教育 OR 课程 OR 项目).ti/ab<br>(prevention OR intervention OR activit* OR education OR curriculum OR program).ti/ab
#4	#1 AND #2 AND #3

Example search terms emerged in the iterative searching stage

Cognitive developmental theory, competence/competency theory, empowerment/empower

(including synonyms)

Appendix B. Summary of critical appraisal (n=62)

Number	Study ID	Intervention	Objectives	Critical appraisal summary
**1**	Alexander (1998)	CARE	This study assessed the efficacy of the C.A.R.E. program on measures of knowledge of CSA prevention concepts, skill acquisition, and rates of disclosure with a population of rural students in Newfoundland. It also sought to provide useful information comparing a shorter term versus a longer term CSAP program. Finally, it addressed some of the underlying assumptions of CSAP programs.	Data collection and analysis thoroughly and conducted; provided. Reasonable level of contextual detail provided; no theoretical frameworks were referenced. Good discussion of how and why findings apply to particular groups.
**2**	Barbee (1992)	Bubbylonian Encounter	This study's research questions are: 1) Is the effectiveness of a sexual abuse prevention program dependent upon the format employed? 2) Is the effectiveness of a sexual abuse prevention program grade-related? 3) Is an increase of knowledge evident after participation in a sexual abuse prevention program? 4) Do students who participate in a sexual abuse prevention program retain the acquired knowledge?	Data collection and analysis reported in the paper; substantial detail on theoretical framework (cognitive development theory).
**3**	Barron & Topping (2010)	Tweenees	This paper presents a detailed analysis of program fidelity within a study of the Tweenees school-based abuse prevention program.	Data collection and analysis conducted and reported; limited information on the theoretical framework that guided the development of the intervention.
**4**	Binder & McNiel (1987)	No name	The study seeks to answer if children's knowledge about sexual abuse prevention increases after participation, if participation causes emotional distress and if parents have an accurate idea of their children's knowledge of sexual abuse prevention.	Data collection and analysis reported. However, contextual information was limited to demographics and locations rather than details directly relating to implementation.
**5**	Blakely, Glaude, & Jennings (2019)	Play it safe	The purpose of this study was to explore school and program factors trainers believed were associated with disclosure among youth.	Provided information on the theoretical framework (trauma-informed framework); context in which program was implemented richly described; factors supported/impeded CSA disclosure discussed; trainers can only ask limited number of questions, but rationale for this is provided. Sample selection and recruitment described adequately.

Appendix B. (continued)

Number	Study ID	Intervention	Objectives	Critical appraisal summary
**6**	Bolen (2003)	N/A	This study compare victim-based paradigms with potential healthy relationship paradigms.	Details on the program mechanism reported; no contextual information provided due to the nature of the study type.
**7**	Briggs & Hawkins (1994a)	Keeping ourselves safe	The study was designed on problem-solving lines to establish whether children could identify and respond safely to a wide range of potentially unsafe situations.	Limitation of the data collection method reported (lack of the rigor of a strong experimental design); contextual information collected and reported.
**8**	Briggs & Hawkins (1994b)	Keeping ourselves safe	This is a follow up study highlighting a developmentally appropriate school-based protection program.	Though the study reported follow-up findings of the program in two countries (Australia and New Zealand), contextual information was limited to demographics and locations rather than details directly relating to implementation.
**9**	Bright, Huq, Patel, Miller, & Finkelhor (2020)	Child Safety Matters	The goal of the curriculum is to educate and empower students to prevent, recognize, and respond appropriately to bullying, cyberbullying, the four types of child abuse, and digital dangers.	Thorough data collection and analytic process; Demographic information collected and reported; limitation of the findings reported ("These results could also be due to the nature of the knowledge itself or failures of the measurement process. Indeed, some of the knowledge tested with the questionnaire had little room for growth.")
**10**	Brown (2017)	Safer smarter kids	This study assessed the effectiveness of the Safer, Smarter Kids kindergarten sexual abuse prevention curriculum in increasing children's knowledge of safety risks and self-protection strategies.	Data collection and analysis reported; no theoretical frameworks discussed; contextual information provided but was limited to the study location rather than details directly relating to implementation.
**11**	Bustamante et al. (2019)	I have the right to feel safe at all times	This study evaluated the immediate and medium-term impact of a 10-week educational program on children's knowledge of CSA self-protection strategies in Ecuador.	Thorough data collection and analytic process; context in which program was implemented richly described (LMICs); candid reflection on limitations of findings.
**12**	Calhoun (2009)	No name	This study examined the relationship between concepts and skills presented in CSAP programs and how effective a facilitator perceived the CSAP program was based on these specific variables.	Theoretical insights (empowerment) provided; data collection and analysis fully described.
**13**	Casper (1998)	Touch Continuum	This study evaluated a sexual abuse prevention program to identify characteristics of children who benefited from the program or became afraid of unwanted touch.	Lack of contextual information provided but was limited to the study location rather than details directly relating to implementation.

Appendix B. (continued)

Number	Study ID	Intervention	Objectives	Critical appraisal summary
**14**	Chen, Zhang, Li, & Luo (2013)	Self-designed curriculum	This study evaluated the impact of school-based child sexual abuse (CSA) prevention education on grade 7 students' knowledge and skills and provide basic information for CSA prevention education.	No information of intervention theory.
**15**	Chen, Huang, He, Wang, Zhang, & Du (2008)	N/A	To understand knowledge, confidence and difficulties of child sexual abuse (CSA) prevention education among child protection workers, and to provide the basis for professional training.	Significant weaknesses in reporting (scant or no information on study design, data collection and analysis) suggest that the overall study design and conduct was weak and that findings should be viewed with caution.
**16**	Czerwinski, Finne, Alfes, & Kolip (2018)	IGEL	To examine the effectiveness of IGEL program in increasing participants' CSA-related knowledge and known courses of action.	Information on implementation process reported; Thorough data collection and analytic process; context in which program was implemented richly described; candid reflection on limitations of findings.
**17**	Daro (1994)	N/A	This paper examines prevention efforts to include public and parent education, life skills training for young adults, support groups for vulnerable children and adults, and intervention for identified victims and perpetrators.	Contextual and theoretical information for CSA prevention discussed; candid reflection on conceptual limitations.
**18**	Zhang & Deng (2019)	Adapted version of Body Safety Training (BST)	This study evaluated the effectiveness of BST program with 92 kindergarten children in Beijing.	Description of program adaptation and factors affecting implementation.
**19**	Finkelhor (2009)	N/A	This study examined initiatives to prevent child sexual abuse, focused on offender management and school-based educational programs.	Theoretical frameworks provided
**20**	Fisher et al. (2005)	self-protection of the handicapped	This article documents the need for a special curricula designed to teach the mentally handicapped self-protection skills to avoid sexual exploitation. In response to this pressing need, a unique and effective curricular approach to this dilemma is offered and detailed.	Lack of details on contextual factors that related to the program implementation process.

Appendix B. (continued)

Number	Study ID	Intervention	Objectives	Critical appraisal summary
**21**	Fryda & Hulme (2015)	N/A	The purpose of this integrative literature review was to determine the state of the science on school-based CSA prevention programs.	Comprehensive review of the literature on CSA; lack of theoretical considerations; limitations reflected.
**22**	Fuqua (2008)	Safe@Last	The purpose of this study was to evaluate the Safe @ Last curriculum by assessing the amount of knowledge that was retained by the participants of the study.	Lack of contextual information provided but was limited to the study location rather than details directly relating to implementation.
**23**	Gibson & Leitenberg (2000)	N/A	The primary goal of the current study was to determine whether rates of child sexual abuse differed among undergraduate women who either had or had not participated in a sexual abuse prevention program during childhood. A secondary goal was to determine whether differences emerged in sexual satisfaction or avoidance of sexual activity between those women who had or had not participated in such a program.	Mechanism of disclosure discussed; clear discussion of the underlying reason for CSA prevention. However, the design is retrospective, subject to recall bias, and the sample is largely Caucasian, and was a convenience sample of undergraduate Psychology students. Some phenomena were also assessed using single item questions.<br><br>Relevance is on the "thin" end of the spectrum, as there is little description of sexual abuse prevention programs – described broadly as good touch/bad touch – presumably to cover a wide range of programs. Explanations presented in Discussion but given sample limitations, generalisability is limited (this is however addressed in Limitations).
**24**	Gushwa, Bernier, & Robinson (2018)	Enough! Preventing CSA in my school	To test the effectiveness of the Enough! Program.	Detailed descriptions of implementation processes; data collection and analysis reported. No detail regarding contextual factors that related to the program implementation process.
**25**	Heidotting (1991)	N/A	The purpose of this study was to investigate the effectiveness of school-based sexual abuse and personal safety prevention programs for children by objectively and empirically synthesizing primary research evidence using the techniques of meta-analysis.	Data collection and analysis reported; lack of reflection on theoretical frameworks and mechanisms.

Appendix B. (continued)

Number	Study ID	Intervention	Objectives	Critical appraisal summary
**26**	Holloway & Pulido (2018)	Safe touches	This article presents data from a CSA prevention program delivered to 2nd and 3rd grade public school children.	Data collection and analysis reported; school context provided. However, theoretical underpinning of Safe Touches was not described at all. There wasn't much discussion of the context and other factors which may have contributed to the results. Relatedly, the program seems to have been adapted to be "culturally appropriate", but details are not provided.
**27**	Barron & Topping (2008)	N/A	This narrative review explored the efficacy of school-based child sexual abuse prevention programs between 1990 and 2002.	Contextual information reported; recommendation provided (e.g. create a supportive school environment; improve teachers' ownership of the program)
**28**	Barron & Topping (2003)	Tweenees	The current exploratory study seeks to address four methodological limitations identified in the literature. These are as follows: the inclusion of high school students, the recording of disclosures within and beyond program lessons, and the introduction of experimental measures of program fidelity and cost.	No theoretical framework provided; No detail regarding contextual factors that related to the program implementation process.
**29**	Jin, Chen, Jiang, & Yu (2017)	Body Safety Training	The aim of this present study was to evaluate the effectiveness of a child sexual abuse (CSA) prevention curriculum toward children and to compare the knowledge gains between children who were taught by teachers and their parents.	Data collection and analysis reported; Cultural contextual factors reported. Lack of reflection on the theoretical framework of the program.
**30**	Kim & Kang (2017)	C-SAPE (Child sexual abuse prevention education program)	The present study aimed to implement the school-based C-SAPE program to empower the children by providing competence in terms of knowledge and self-protective behaviors related to CSA. Its secondary goal was to evaluate the effectiveness of the C-SAPE.	The iterative development of the intervention is closely described. Cleary description of the theoretical framework. Sufficient information regarding implementation context.

Appendix B. (continued)

Number	Study ID	Intervention	Objectives	Critical appraisal summary
**31**	Ko (2001)	Abuse prevention	The present study examines the relationship between attendance at prevention programs and subsequent knowledge and experience of abuse through a self-report questionnaire created by the researcher.	Data collection and analysis reported; rational of the study and program provided; No detail regarding contextual factors that related to the program implementation process.
**32**	Kolko (1988)	N/A	This paper provides an overview and critique of various prevention programs conducted in school and community settings, highlighting their achievements and limitations. The methods, characteristics, and empirical outcomes of specific programs are discussed along with future directions and recommendations.	Theoretical framework provided; sufficient information regarding contextual factors at different levels.
**33**	Kolko, Moser, Litz, & Hughes (1987)	Red flag green flag and presentation of a film called better safe than sorry ii	The objectives of the Pittsburgh Red Flag/Green Flag Program were to assist children in developing the vocabulary needed to report cases of abuse to a trusted adult, to expedite reporting and investigation of individual cases of child abuse, and to teach children to say "no" to potential abusers and physically "get away" from potentially harmful situations.<br><br>The ultimate goal of this program was to begin to reduce the incidence of child sexual abuse and victimization in Washington County, Pennsylvania.	Mechanisms of the program reported; consideration of the context within which the intervention was implemented.
**34**	Kraizer, Witte, & Fryer (1989)	Safe children	To evaluate the Safe Child program.	Lack of reflection on the theoretical framework of the program.

Appendix B. (continued)

Number	Study ID	Intervention	Objectives	Critical appraisal summary
**35**	Krivacska (1993)	The PRISM program (Prevention, Reduction and Identification of Sexual Misuse)	To review of a CSA prevention program (PRISM).	Rationale of the program components clearly described (e.g. the social solving component). Contextual factors were described.
**36**	Lape (1999)	The Sexual Abuse Prevention Project for Preschool/ Kindergartners	The present study sought to determine if preschool children could learn sexual abuse prevention concepts, if parents could learn sexual abuse information from a parent meeting, if parents' attitude change as a result of attending the meeting, and if the amount a preschool child benefits from a sexual abuse prevention program is related to parent knowledge gain or attitude change.	Rationale of the outcome measurement described. Discussion was helpfully tailored around implications for preschool children, alongside parental involvement and its impact on outcomes.
**37**	Lee & Ju (2016)	Sexual abuse Prevention Education connected with Physical and Psychological Development Education Program	The purpose of this study was to investigate the effects of sexual abuse prevention education program linked to elementary physical and psychological development on sexual knowledge and attitude.	Data collection and analysis clearly reported; no sufficient information regarding the intervention theory.
**38**	Madak & Berg (1992)	Talking about touching	To examine the effectiveness of a CSA prevention program, 'Talking About Touching".	Data collection and analysis reported; no information on the intervention theory and the context that related to the program implementation. Schools were not randomly chosen, so some impact on sampling and bias. Seems like instruments were developed specifically for the study and were not previously validated. Overall sample size is low for the kind of questions it seeks to answer. Pre-

				testing may also have affected results. Mainly descriptive analyses.

Appendix B. (continued)

**Number**	**Study ID**	**Intervention**	**Objectives**	**Critical appraisal summary**
**39**	Manheim, Felicetti, & Moloney (2019)	N/A	This paper will review the literature that provides the rationale behind offering these types programs to the youngest of school-children, what these programs can offer, and describe specific programs that have been validated for preschool and kindergarten-aged children, using the best practice guidelines in the field of early childhood education.	Clear description of the theoretical framework and mechanism underpinning the intervention.
**40**	Moon, Park, & Sung (2017)	SAP_MobAPP	This study aimed to develop and evaluate the effects of a sexual abuse prevention mobile application, SAP_MobAPP, for primary school children.	The iterative development of the intervention is closely described. Cleary description of the theoretical framework. Sufficient information regarding implementation context.
**41**	Neherta, Machmud, Damayanti, & Afrizal (2017)	Visual Auditory Kinesthetic modalities	To determine the effectiveness of interventions for sexual abuse prevention on knowledge and assertiveness behavior of primary school age children in Padang, which were committed by two different professions, namely nurses and teachers.	Data collection and analysis reported; lack of information on the intervention theory and the context that related to the program implementation process.
**42**	Nurdin, Neherta, & Meri (2018)	Neherta	To know the effectiveness intervention module of sexual abuse prevention against Children from “Neherta” model	Lack of discussion of the context that related to the program implementation process; lack of reflection on the study limitations.
**43**	Pelcovitz, Adler, Kaplan, Packman, & Krieger (1992)	a brief, one session, not targeted at different age group	The current study presents the results of interviews with a group of child victims of extrafamilial sexual abuse who, in spite of seeing a sexual abuse prevention film during the period they were being abused, did not disclose that they were being sexually molested.	No information on the intervention theory; no discussion on contextual factors related to the program implementation process.
**44**	Rispens, Aleman, & Goudena (1997)	N/A	The aim of this article was to provide data about the effects of child sexual abuse prevention programs. A more specific aim was to estimate the contribution of potential moderator variables such as age, program duration, or sample size to effect	Data collection and analysis reported; moderator analysis conducted to provide insights on the program effect.

size.

Appendix B. (continued)

Number	Study ID	Intervention	Objectives	Critical appraisal summary
**45**	Ruldoph & Zimmer-Gembeck (2018)	N/A	This paper summarizes the recent history of CSA prevention and the critique of child-focused protection programs in order to demonstrate the need to compliment or replace these programs by focusing more on protectors in the children's ecology, specifically parents, in order to create safer environments in which abuse is less likely to occur.	Clear description of the outcome mechanism (disclosure); discussion of the underpinning theoretical framework of the programs.
**46**	Russell, Higgins, & Posso (2020)	N/A	To systematically review the available evidence on the effectiveness of CSA prevention strategies in developing countries. The research question that guided the analysis is: What type of sexual abuse prevention interventions or initiatives are being implemented in developing countries, and are they effective?	Clear description of the cultural context; mechanisms discussed.
**47**	Scholes, Jones, Stieler-Hunt, Rolfe, & Pozzebon (2012)	N/A	First, the paper examines concerns about the lack of explicit professional development for educators concerning child protection, and the need to develop understandings about prevention program best practices within pre-service and in-service training. Second, drawing on a systematic review of literature, the paper identifies five key considerations to inform teachers' selection and facilitation of CSA prevention curriculum in school contexts. Third, the paper advances calls by Wurtele (2009) and presents CSA prevention 'best practices' overview and 'model programs' list for professionals such as teachers.	Demographic differences discussed; Methods are described less well, especially given that this is a systematic review. Implications of key considerations are not discussed in any great detail at all, although the findings offer useful information and summary of existing research; limitation reflected; lack of information regarding program theory.
**48**	Taal & Edelaar (1997)	Right to security	This study evaluated a sexual abuse prevention program for sixth, seventh, and eighth graders ranging from 8 to 12 years of age.	Details of program development described; lack of description of the context within which the intervention took place.

Appendix B. (continued)

Number	Study ID	Intervention	Objectives	Critical appraisal summary
**49**	Telljohann, Everett, & Price (1997)	Sexual Abuse Prevention Program, Third Grade Curriculum	This paper presents results from a third grade sexual abuse prevention program. A 24-item questionnaire was administered as a pretest and post-test to an experimental group (n = 236) and a control group (n = 195) of third grade students.	Data collection and analysis reported; no reflections on the intervention program theory and the context in which the intervention took place.
**50**	Trudell & Whatley (1988)	N/A	This article critically examines current assumptions about the role of elementary school personnel in prevention and possible unintended consequences of such assumptions.	Discussion and description of the factors affecting implementation (fidelity e.g. assessment).
**51**	Tutty (1994)	Touching	This paper reviews the literature debating the appropriateness of providing child sexual abuse prevention programs to young children.	Discussion of the program theory; discussion of factors affecting implementation and program effects.
**52**	Tutty (2000)	Who do you tell?	This article describes the influence of development on whether children learn certain child abuse prevention concepts, reviewing the small body of literature about previously conducted item analyses on changes in children's knowledge levels after participation in a child sexual abuse prevention program.	Discussion of factors affecting implementation and program effects.
**53**	Tutty (2014)	Who do you tell?	This article presents the results of qualitative research with 116 students (51 boys and 65 girls) ranging from age 6 to 12 who had participated in the Who Do You Tell child sexual abuse education program.	Data collection and analysis reported; findings are closely linked to the interview data; limitations reflected.
**54**	Tutty, Aubry, & Velasquez (2020)	Who do you tell?	This article presents the results of subsequently monitoring program outcomes over an eight-year period (2010–2017), examining knowledge/attitudes in 6198 students in 50 schools in a Western Canadian city.	Discussion of factors affecting implementation and program effects.

Appendix B. (continued)

Number	Study ID	Intervention	Objectives	Critical appraisal summary
**55**	Wood & Archbold (2015)	Red flag green flag people	To examine the efficacy of the “Red Flag Green Flag People” program presented to elementary school children in two school districts in the Midwest.	Intervention is described in detail; Discussion of factors affecting implementation and program effects; lack of description of intervention theory; sample is majority white. But sampling strategy and survey procedures are well described; Findings are described for specific groups and reasons are offered as to why.
**56**	Wurtele (1987)	N/A	In this paper, programs which have been implemented in grades K-6 are summarized according to mode and content of presentation, audience and trainer characteristics, and program length.	Discussion of factors affecting implementation and program effects.
**57**	Wurtele, Kast, & Melzer (1992)	behavioral skills training	To compare teachers and parents as instructors of a personal safety program.	Descriptions of program theory and rationale of the program components were provided.
**58**	Wurtele (1998)	N/A	To present and address some of concerns about CSA prevention programs.	Discussion of factors affecting implementation and program effects.
**59**	Xie, Qiao, & Miller (2020)	N/A	To explore myths about CSA in the Chinese cultural contexts and its reasons. The current study will provide evidence for the development of CSA prevention curricula.	Contextual factors described; data was reported in little detail.
**60**	Yang (2019)	N/A	This article reviewed the theoretical basis, basic principles and approaches to prevent CSA in current Chinese society.	Contextual factors described; lack of descriptions of the theoretical underpinning.
**61**	Yi, Zhao, Qiu, & Wang (2020)	N/A	This paper proposes countermeasures from four aspects of school, family, society and the linkage of the three, aiming to provide theoretical references for the construction of curriculum and practice education in primary schools about CSA prevention education.	Lack of description of intervention theory; descriptions of contextual factors.
**62**	Yom and Eun (2005)	CD Rom	To test the effectiveness of a sexual violence prevention programs in a middle school.	Descriptions of the program development process; no description of intervention theory.

Appendix C. Studies that used for the development of initial program theory (n=44)

Number	Study ID	Country	Study design/methods	Intervention	Objectives
**1**	Ages (1991)	Canada	Quantitative: pre- and post-test study design	Personal Safety Program	This study evaluated the maintenance of changes in information and behavioral skills effected by a school-based personal safety program.
**2**	Alexander (1998)	Canada	Quantitative: quasi-experimental	Child Abuse Research and Education (CARE)	The purpose of this study was threefold. Firstly, the study assessed the efficacy of the C.A.R.E. program. Secondly, it sought to provide useful information comparing a shorter term versus a longer term CSAP program through follow-up of grade six students who previously had received either program, up to grade three or grade five. Finally, it addressed some of the underlying assumptions of CSAP programs.
**3**	Araji, Fenton, & Straugh (1995)	USA	Quantitative: pre-and post-test study	Teaching about Touching	This article describes and evaluates a K-6 child sexual abuse prevention curriculum that was piloted in a large northwestern school district 1986-89.
**4**	Bae & Panuncio (2009)	South Korea	Mixed methods: qualitative and quantitative	Computer-based CSA prevention program	This study describes the development of an interactive, educational computer-assisted instruction (CAI) program using a multimedia CD-ROM for child sexual abuse prevention.
**5**	Baker, Gleason, Naai, Mitchell, & Trecker (2013)	USA	Quantitative: quasi-experimental	My Body My Boundaries	This preliminary study evaluates changes in children’s knowledge of sexual abuse using a school-based train-the-trainer curriculum.

Appendix C. (continued)

Number	Study ID	Country	Study design/methods	Intervention	Objectives
**6**	Barron & Topping (2013)	UK	Quantitative: quasi-experimental	Tweenees	The current exploratory study seeks to address four methodological limitations identified in the literature. These are as follows: the inclusion of high school students, the recoding of disclosures within and beyond program lessons, and the introduction of experimental measures of program fidelity and cost.
**7**	Blumberg, Chadwick, Fogarty, Speth, & Chadwick (1991)	USA	Quantitative: cluster RCT	Role play program versus Multimedia program	The purpose of this study was to examine the effectiveness of two prevention programs in improving children's abilities to discriminate between appropriate and in appropriate types of touching.
**8**	Briggs & Hawkins (1994)	Australia	Quantitative: questionnaire	Keeping Ourselves Safe	The aim of the study was to offer quantitative support for the continued teaching of the Keeping Ourselves Safe program.
**9**	Casper (1999)	USA	Quantitative: pre- and post-test study	Touch Continuum	This study evaluated a sexual abuse prevention program to identify characteristics of children who benefited from the program or became afraid of unwanted touch.
**10**	Chen, Fortson, & Tseng (2012)	Taiwan	Quantitative: RCT	A CSA prevention program developed based on behavioral skills training	The purpose of the current study was to develop and evaluate the efficacy of a school-based child sexual abuse prevention program for Taiwanese children.

Appendix C. (continued)

Number	Study ID	Country	Study design/methods	Intervention	Objectives
**11**	Counts (2003)	USA	Quantitative: controlled before-and-after study design	Smart Kids Safe Kids	A study was conducted to evaluate the amount of knowledge retained by students participating in the Smart Kids/Safe K ids sexual abuse prevention program.
**12**	Crowley (1989)	USA	Quantitative: quasi-experimental	Good Touches/Bad Touches	This study evaluated the effectiveness of the Good Touches/Bad Touches (GT/BT) program.
**13**	Daigneault, Hébert, McDuff, & Frappier (2012)	Canada	Quantitative: quasi-experimental	ESCAPE CSA prevention workshop	The goal of the present study was to evaluate the effectiveness of the ESPACE sexual abuse prevention workshop.
**14**	Dake, Price, & Murnan (2003)	USA	Quantitative: cluster RCT	No name (modified version of an existing CSA prevention program)	Researchers investigated the effects of a child abuse prevention program with third-grade students.
**15**	Dhooper & Schneider (1995)	USA	Quantitative: controlled before-and-after study design/quasi-experimental design	School-based child abuse prevention program	This article reports the results of an evaluation study of a school-based educational child abuse prevention program using a quasi-experimental design.
**16**	Fryer, Kraizer, & Mlyoshi (1987)	USA	Quantitative: RCT	Body Safety Training; film “Touch”	We compared the effectiveness of various educational approaches for teaching personal safety skills to children in an effort to possibly prevent sexual abuse.

Appendix C. (continued)

**Number**	**Study ID**	**Country**	**Study design/methods**	**Intervention**	**Objectives**
**17**	Grendel (1991)	USA	Quantitative: wait list control	CSA prevention program by Women Helping Women	The purpose of this study was to examine the cognitive and emotional effects of a brief CSA prevention program for first graders.
**18**	Harvey, Forehand, Brown, & Holmes (1988)	USA	Quantitative: RCT	Good Touches/Bad Touches	The purpose of the present study was to evaluate a program designed to teach skills to young children in order to prevent CSA.
**19**	Hazzard, Webb, Kleemeier, Angert, & Pohl (1991)	USA	Quantitative: cluster RCT	Feeling Yes Feeling No	In this multimodal study, a 3-session adaptation of the Feeling Yes, Feeling No curriculum was provided to 286 third and fourth graders from four schools whose responses were compared to 113 delayed-treatment control children from two schools.
**20**	Hébert, Lavoie, Piché, & Poitras, (2001)	Canada	Quantitative: quasi-experimental	ESCAPE	The effects of the sexual child abuse prevention program ESPACE were evaluated in the study.
**21**	Herman (1987)	USA	Quantitative: pre-test and post-teat with multiple baseline study	My Very Own Book about Me	This study evaluated the effectiveness of a district-wide sex abuse curriculum prevention program using My Very Own Book About Me.
**22**	Jin, Chen, Jiang, & Yu(2017)	China	Quantitative: Cluster RCT	Body Safety Training	The aim of this present study was to evaluate the effectiveness of a child sexual abuse (CSA) prevention curriculum.

Appendix C. (continued)

Number	Study ID	Country	Study design/methods	Intervention	Objectives
**23**	Kolko, Moser, & Hughes (1989)	USA	Quantitative: cluster RCT	Red Flag/Green Flag	This study is an extension of a previous evaluation of a program (Red Flag/Green Flag) in which children, parents, and teachers were exposed to a workbook and film designed to teach personal safety strategies for preventing sexual victimization or encouraging adult assistance through disclosure of such incidents.
**24**	Kolko, Moser, Litz, & Hughes (1987)	USA	Quantitative: controlled before-and-after study design	Red Flag/Green Flag; Better Safe than Sorry (Film)	This study describes an evaluation of a school-based sexual abuse awareness and prevention program that featured the "Red Flag/Green Flag People" coloring book and included presentation of a film ("Better Safe than Sorry") and discussion of hypothetical and actual experiences involving inappropriate physical touching.
**25**	Kraizer (1991)	USA	Quantitative: cluster RCT	The Safe Child Program	This Ph.D. project has included the development and formative evaluation of The Safe Child Program.
**26**	Lee & Tang (1998)	China	Quantitative: RCT	Behavioral Skills Training	The aim of the study was to evaluate the effectiveness of BST program.

Appendix C. (continued)

Number	Study ID	Country	Study design/methods	Intervention	Objectives
**27**	Macintyre & Carr (1999a)	Ireland	Quantitative: Cross-sectional comparative study	Stay Safe Program	This study aimed to evaluate the effectiveness of a school-based safety skills program—The Stay Safe Program.
**28**	Madak & Berg (1992)	Canada	Quantitative: pre- and post-test study	Talking about Touching	This study was conducted to evaluate the program Talking About Touching.
**29**	Morris et al., (2017)	USA	Quantitative: cluster RCT	Safe@Last	The primary aim of the present study was to examine two community-level characteristics as potential moderators of the Safe@Last program effectiveness.
**30**	Müller, Röder, & Fingerle (2014)	Germany	Quantitative: wait list control	Cool and Safe	The present contribution introduces a newly developed web-based training aiming at the prevention of child sexual abuse and describes the results of the first evaluation of “Cool and Safe”.
**31**	Oldfield, Hays, & Megel (1996)	USA	Quantitative: cluster RCT	Project TRUST	This study assessed the effects of Project TRUST.
**32**	Poche, Yoder, & Miltenberger (1988)	USA	Quantitative: cluster RCT	Videotape	This study compared the effectiveness of a videotape training program with other methods of teaching children self-protection to prevent child abduction.
**33**	Saslawsky & Wurtele (1986)	USA	Quantitative: quasi-experimental	Film “Touch”	We compared the effectiveness of various educational approaches for teaching personal safety skills to children.

Appendix C. (continued)

Number	Study ID	Country	Study design/methods	Intervention	Objectives
**34**	Smothers & Smothers (2011)	USA	Quantitative: pre- and post-test study design	No Name	The goal of the program was to promote and create community change within individuals and the school community by reducing tolerance of sexual violence and sexual harassment.
**35**	Snyder (1986)	USA	Quantitative: quasi-experimental	Good Secret Bad Secret	The purpose of this study was to evaluate the effectiveness of the "Good Secrets, Bad Secrets" program.
**36**	Taal & Edelaar (1997)	The Netherlands	Quantitative: controlled before-and-after study design	Right to Security	This study evaluated the effectiveness of a sexual abuse prevention program.
**37**	Telljohann, Everett, & Price (1997)	USA	Quantitative: controlled before-and-after study design	sexual abuse prevention program third program third grade curriculum	This paper presents results from a third-grade sexual abuse prevention program.
**38**	Tutty (1997)	Canada	Quantitative: RCT	Who Do You Tell?	The research evaluated a sexual abuse prevention program for elementary school-aged children.
**39**	Tutty (1991)	Canada	Quantitative: quasi-experimental	Touching	The current research was designed to investigate a wide range of variables which could affect the ability of elementary school children to learn and to remember child sexual abuse prevention concepts.
**40**	Volpe (1984)	Canada	Quantitative: post-test only	The child abuse component of human relations program	This paper reports the outcome of an attempt to teach children in grades 5 and 6 about child abuse and neglect.

Appendix C. (continued)

Number	Study ID	Country	Study design/methods	Intervention	Objectives
**41**	Warden, Moran, Gillies, Mayes, & Macleod (1997)	UK	Quantitative: controlled before and after study design	Kidscape	This paper reports an evaluation of a children's safety training program, Kidscape.
**42**	Weatherley et al. (2012)	Malaysia	Quantitative: pre- post-test	Keeping me safe	This article examined the effectiveness of a personal safety curriculum (Keeping Me Safe) offered to Standard Three students (mostly nine-year-olds).
**43**	Wolfe, MacPherson, Blount, & Wolfe (1986)	USA	Quantitative: quasi-experimental	play	The present study was conducted to evaluate proximal changes in fourth and fifth graders' knowledge and attitudes of physical and sexual abuse following brief skits and focal discussion in the classroom.
**44**	Wurtele, Saslawsky, Miller, Marrs, & Britcher (1986)	USA	Quantitative: RCT	Body Safety Training; Film called "Touch"	We tested the prediction that a sexual abuse prevention program that included participant modeling (PM) would result in superior skill acquisition compared with a symbolic modeling (SM) program.

Appendix D. Studies that were coded to develop the refined program theory (CSMOs) (n=62)

Number	Study ID	Country	Study design/methods	Intervention	Objectives	Population
**1**	Alexander (1998)	Canada	Quantitative	CARE	The purpose of this study was threefold. Firstly, the study assessed the efficacy of the C.A.R.E. Secondly, it sought to provide useful information comparing a shorter term versus a longer term CSAP program through follow-up of grade six students who previously had received either program, up to grade three or grade five. Finally, it addressed some of the underlying assumptions of CSAP programs.	k-5th grade
**2**	Barbee (1992)	USA	Quantitative	Bubbylonian Encounter	The research questions are: 1) Is the effectiveness of a sexual abuse prevention program dependent upon the format (i.e., type of medium employed)? 2) Is the effectiveness of a sexual abuse prevention program grade-related? 3) Is an increase of knowledge evident after participation in a sexual abuse prevention program? 4) Do students who participate in a sexual abuse prevention program retain the acquired knowledge?	k-6th grade
**3**	Barron & Topping (2010)	UK	Process evaluation	Tweenees	This paper identifies key themes from research into program fidelity of sexual abuse prevention programs.	Secondary school students

Appendix D. (continued)

Number	Study ID	Country	Study design/methods	Intervention	Objectives	Population
**4**	Binder & McNiel (1987)	USA	Quantitative	No name	The study seeks to answer: 1) Does children's knowledge about sexual abuse prevention increase after participation in the program? 2) Do parents have an accurate idea of their children's knowledge regarding sexual abuse prevention prior to and after the program? 3) Does the sexual abuse prevention program cause emotional distress in children?	Children aged 5-12
**5**	Blakely, Glaude, & Jennings (2019)	USA	Qualitative	Play it safe	The purpose of this study was to explore school and program factors that trainers in a school-based prevention program believed were associated with disclosure among youth from kindergarten through 12th grade.	Trainers and program administrators
**6**	Bolen (2003)	USA	Commentary	N/A	The article compares the existing victim-based paradigm with the proposed potential healthy relationships paradigms.	N/A
**7**	Briggs & Hawkins (1994a)	New Zealand	Quantitative	Keeping ourselves safe	The interview schedule was designed on problem-solving lines to establish whether children could identify and respond safely to a wide range of potentially unsafe situations.	Primary school students aged 6-9
**8**	Briggs & Hawkins (1994b)	New Zealand; Australia	Quantitative	Keeping ourselves safe	The present paper reports on a follow up study which reveals the importance of providing young children with a school-based protection program which is developmentally appropriate in terms of language and concepts.	Primary school students aged 5-8
**9**	Bright, Huq, Patel, Miller, & Finkelhor (2020)	USA	Quantitative	Child Safety Matters	The goal of the curriculum is to educate and empower students to prevent, recognize, and respond appropriately to bullying, cyberbullying, the four types of child abuse, and digital dangers.	k-5th

Appendix D. (continued)

Number	Study ID	Country	Study design/methods	Intervention	Objectives	Population
**10**	Brown (2017)	USA	Quantitative	Safer smarter kids	This study assessed the effectiveness of the Safer, Smarter Kids kindergarten sexual abuse prevention curriculum.	5.5-7.7 years old
**11**	Bustamante et al. (2019)	Ecuador	Quantitative	I have the right to feel safe at all times	To evaluate the immediate and medium-term impact of a 10-week educational program on children's knowledge of CSA self-protection strategies in Ecuador.	7-12 years old
**12**	Calhoun (2009)	USA	Quantitative	No name	This study examined the relationship between concepts and skills presented in CSAP programs and how effective a facilitator perceived the CSAP program was based on these specific variables.	School counselors
**13**	Casper (1998)	USA	Quantitative	Touch Continuum	This study evaluated a sexual abuse prevention program to identify characteristics of children who benefited from the program or became afraid of unwanted touch.	2-6th graders
**14**	Chen, Zhang, Li, & Luo (2013)	China	Quantitative: Pre- and post-test design	Self-designed curriculum	To evaluate the impact of school-based child sexual abuse (CSA) prevention education.	7th graders
**15**	Chen, Huang, He, Wang, Zhang, & Du (2008)	China	Quantitative: questionnaire	N/A	To understand knowledge, confidence and difficulties of child sexual abuse (CSA) prevention education among child protection workers, and to provide the basis for professional training.	Child protection workers
**16**	Czerwinski, Finne, Alfes, & Kolip (2018)	Germany	Quantitative	IGEL	To examine the effectiveness of IGEL program.	3rd grade students

Appendix D. (continued)

Number	Study ID	Country	Study design/methods	Intervention	Objectives	Population
**17**	Daro (1994)	USA	Critique	N/A	The article concludes with suggestions for expanding prevention efforts to include public and parent education, life skills training for young adults, support groups for vulnerable children and adults, and intervention for identified victims and perpetrators.	N/A
**18**	Zhang & Deng (2019)	China	Quantitative: RCT	Adapted version of Body Safety Training (BST)	The study evaluated the effectiveness of BST program.	
**19**	Finkelhor (2009)	USA	Review	N/A	To examine initiatives to prevent child sexual abuse, which have focused on two primary strategies- offender management and school-based educational programs.	
**20**	Fisher et al. (2005)	USA	Quantitative	self-protection of the handicapped	This article documents the need for a special curricula designed to teach the mentally handicapped self-protection skills to avoid sexual exploitation.	Mentally handicapped students
**21**	Fryda & Hulme (2015)	USA	Review	N/A	The purpose of this integrative literature review was to determine the state of the science on school-based CSA prevention programs.	N/A
**22**	Fuqua (2008)	USA	Quantitative	Safe@Last	The purpose of this study was to evaluate the Safe @ Last curriculum by assessing the amount of knowledge that was retained by the participants of the study.	1st - 4th graders
**23**	Gibson & Leitenberg (2000)	USA	Quantitative	N/A	The primary goal of the current study was to determine whether rates of child sexual abuse differed among undergraduate women who either had or had not participated in a sexual abuse prevention program during childhood.	College female students

Appendix D. (continued)

Number	Study ID	Country	Study design/methods	Intervention	Objectives	Population
**24**	Gushwa, Bernier, & Robinson (2018)	USA	Quantitative	Enough! Preventing CSA in my school	To test the effectiveness of the Enough! Program.	School teachers and personnel
**25**	Heidotting (1991)	USA	Review	N/A	The purpose of this study was to investigate the effectiveness of school-based sexual abuse and personal safety prevention programs.	
**26**	Holloway & Pulido (2018)	USA	Quantitative	Safe touches	This article presents data from a CSA prevention program delivered to 2nd and 3rd grade public school children.	2nd-3rd graders
**27**	Barron & Topping (2008)	USA	Review	N/A	This narrative review explored the efficacy of school-based child sexual abuse prevention programs between 1990 and 2002.	N/A
**28**	Barron & Topping (2003)	UK	Quantitative	Tweenees	The current exploratory study seeks to address four methodological limitations identified in the literature. These are as follows: the inclusion of high school students, the recording of disclosures within and beyond program lessons, and the introduction of experimental measures of program fidelity and cost.	Grade 6-8th; average age 12.55
**29**	Jin, Chen, Jiang, & Yu (2017)	China	Quantitative: Cluster RCT	Body Safety Training	The aim of this present study was to evaluate the effectiveness of a child sexual abuse (CSA) prevention curriculum toward children and to compare the knowledge gains between children who were taught by teachers and their parents.	

Appendix D. (continued)

Number	Study ID	Country	Study design/methods	Intervention	Objectives	Population
**30**	Kim & Kang (2017)	South Korea	Quantitative	C-SAPE (Child sexual abuse prevention education program)	The present study aimed to implement the school-based C-SAPE program to empower the children by providing competence in terms of knowledge and self-protective behaviors related to CSA. Its secondary goal was to evaluate the effectiveness of the C-SAPE.	5th grade students
**31**	Ko (2001)	USA	Quantitative	Abuse prevention	The present study examines the relationship between attendance at prevention programs and subsequent knowledge and experience of abuse through a self-report questionnaire created by the researcher.	High school students
**32**	Kolko (1988)	USA	Review	N/A	This paper provides an overview and critique of various prevention programs conducted in school and community settings, highlighting their achievements and limitations.	N/A
**33**	Kolko, Moser, Litz, & Hughes (1987)	USA	Quantitative	Red flag green flag and presentation of a film called better safe than sorry ii	The objectives of the Pittsburgh Red Flag/Green Flag Program were to assist children in developing the vocabulary needed to report cases of abuse to a trusted adult, to expedite reporting and investigation of individual cases of child abuse, and to teach children to say "no" to potential abusers and physically "get away" from potentially harmful situations.	Children parents and teachers
**34**	Kraizer, Witte, & Fryer (1989)	USA	Newspaper	Safe children	To evaluate the Safe Child program.	3-10 years olds

Appendix D. (continued)

Number	Study ID	Country	Study design/methods	Intervention	Objectives	Population
**36**	Lape (1999)	USA	Quantitative	The Sexual Abuse Prevention Project for Preschool/ Kindergartners	The present study sought to determine if preschool children could learn sexual abuse prevention concepts, if parents could learn sexual abuse information from a parent meeting, if parents' attitude change as a result of attending the meeting, and if the amount a preschool child benefits from a sexual abuse prevention program is related to parent knowledge gain or attitude change.	Preschoolers and parents
**37**	Lee & Ju (2016)	South Korea	Quantitative	Sexual abuse Prevention Education connected with Physical and Psychological Development Education Program	The purpose of this study was to investigate the effects of sexual abuse prevention education program.	5th-6th graders
**38**	Madak & Berg (1992)	Canada	Quantitative	Talking about touching	To examine the effectiveness of a CSA prevention program, 'Talking About Touching".	k-6th; children parents and teachers
**39**	Manheim, Felicetti, & Moloney (2019)	USA	Review	N/A	This paper will review the literature that provides the rationale behind offering these types programs to the youngest of school-children.	Preschoolers
**40**	Moon, Park, & Sung (2017)	South Korea	Quantitative	SAP_MobAPP	This study aimed to develop and evaluate the effects of a sexual abuse prevention mobile application, SAP_MobAPP, for primary school children.	10 years-old

Appendix D. (continued)

Number	Study ID	Country	Study design/methods	Intervention	Objectives	Population
**41**	Neherta, Machmud, Damayanti, & Afrizal (2017)	Indonesia	Quantitative	Visual Auditory Kinesthetic modalities	To determine the effectiveness of interventions for sexual abuse prevention on knowledge and assertiveness behavior of primary school age children in Padang.	9.5 years-old
**42**	Nurdin, Neherta, & Meri (2018)	Indonesia	Quantitative	Neherta	To know the effectiveness intervention Module of sexual abuse prevention against Children from “Neherta” model.	3-5th graders
**43**	Pelcovitz, Adler, Kaplan, Packman, & Krieger (1992)	USA	Qualitative	a brief, one session, not targeted at different age group	The current study presents the results of interviews with a group of child victims of extrafamilial sexual abuse.	6-10 years olds
**44**	Rispens, Aleman, & Goudena (1997)	USA	Meta-analysis	N/A	The aim of this article was to provide data about the effects of child sexual abuse prevention programs.	N/A
**45**	Ruldoph & Zimmer-Gembeck (2018)	USA	Review	N/A	This paper summarizes the recent history of CSA prevention and the critique of child-focused protection programs.	N/A
**46**	Russell, Higgins, & Posso (2020)	N/A	Systematic review	N/A	To systematically review the available evidence on the effectiveness of CSA prevention strategies in developing countries.	Most empirically evaluated interventions in developing countries have focused on preschool and primary school-aged children.

Appendix D. (continued)

Number	Study ID	Country	Study design/methods	Intervention	Objectives	Population
**47**	Scholes, Jones, Stieler-Hunt, Rolfe, & Pozzebon (2012)	Australia	Review	N/A	First, the paper examines concerns about the lack of explicit professional development for educators concerning child protection, and the need to develop understandings about prevention program best practices within pre-service and in-service training. Second, drawing on a systematic review of literature, the paper identifies five key considerations to inform teachers' selection and facilitation of CSA prevention curriculum in school contexts. Third, the paper advances calls by Wurtele (2009) and presents CSA prevention 'best practices' overview and 'model programs' list for professionals such as teachers.	N/A
**48**	Taal & Edelaar (1997)	The Netherlands	Quantitative	right to security	This study evaluated a sexual abuse prevention program for sixth, seventh, and eighth graders ranging from 8 to 12 years of age.	8-12 years olds
**49**	Telljohann, Everett, & Price (1997)	USA	Quantitative	Sexual Abuse Prevention Program, Third Grade Curriculum	This paper presents results from a third grade sexual abuse prevention program.	9 years old

Appendix D. (continued)

Number	Study ID	Country	Study design/methods	Intervention	Objectives	Population
**50**	Trudell & Whatley (1988)	USA	Review	N/A	This article critically examines current assumptions about the role of elementary school personnel in prevention and possible unintended consequences of such assumptions.	N/A
**51**	Tutty (1994)	Canada	Review	Touching	This paper reviews the literature debating the appropriateness of providing child sexual abuse prevention programs to young children.	1st, 3rd, 6th graders
**52**	Tutty (2000)	Canada	Quantitative	Who do you tell?	This article describes the influence of development on whether children learn certain child abuse prevention concepts.	N/A
**53**	Tutty (2014)	Canada	Qualitative	Who do you tell?	This article presents the results of qualitative research with 116 students who had participated in the Who Do You Tell child sexual abuse education program.	6-12 years olds
**54**	Tutty, Aubry, & Velasquez (2020)	Canada	Quantitative	Who do you tell?	This article presents the results of subsequently monitoring program outcomes over an eight-year period (2010–2017).	Elementary school students
**55**	Wood & Archbold (2015)	USA	Quantitative	Red flag green flag people	To examine the efficacy of the “Red Flag Green Flag People” program presented to elementary school children in two school districts in the Midwest.	3rd and 4th grade students
**56**	Wurtele (1987)	USA	Review	N/A	In this paper, programs which have been implemented in grades K-6 are summarized according to mode and content of presentation, audience and trainer characteristics, and program length.	N/A

Appendix D. (continued)

Number	Study ID	Country	Study design/methods	Intervention	Objectives	Population
**57**	Wurtele, Kast, & Melzer (1992)	USA	Quantitative	behavioral skills training	To compare teachers and parents as instructors of a personal safety program.	Preschoolers
**58**	Wurtele (1998)	USA	Book chapter	N/A	To present and address some of concerns about CSA prevention programs.	N/A
**59**	Xie, Qiao, & Miller (2020)	China	Mixed methods	N/A	To explore myths about CSA in the Chinese cultural contexts and its reasons. The current study will provide evidence for the development of CSA prevention curricula.	
**60**	Yang (2019)	China	Review	N/A	This article reviewed the theoretical basis, basic principles and approaches to prevent CSA in current Chinese society.	
**61**	Yi, Zhao, Qiu, & Wang (2020)	China	Review	N/A	This paper proposes countermeasures from four aspects of school, family, society and the linkage of the three, aiming to provide theoretical references for the construction of curriculum and practice education in primary schools about CSA prevention education.	
**62**	Yom and Eun (2005)	South Korea	Quantitative	CD Rom	To test the effectiveness of a sexual violence prevention programs in a middle school.	Male middle school students

Appendix E. List of studies that are included in the review but not referenced in the manuscript

Ages, S. E. (1991). *An evaluation of the maintenance of changes in knowledge and behaviour skills effected by a school-based personal safety program for the primary grades* (Doctoral dissertation, Carleton University).

Araji, S. K., Fenton, R., & Straugh, T. (1995). Child sexual abuse: Description and evaluation of a K-6 prevention curriculum. *Journal of Primary Prevention*, *16*(2), 149-164.

Bae, J., & Panuncio, R. L. (2009). Development of Computer-Assisted Instruction Program for Child Sexual Abuse Prevention. *International Journal of Computer Science and Network Security*, *9*(3), 142-7.

Briggs, F., & Hawkins, R. M. (1996). A comparison of the childhood experiences of convicted male child molesters and men who were sexually abused in childhood and claimed to be nonoffenders. *Child abuse & neglect*, *20*(3), 221-233.

Counts, M. A. (2003). Smart Kids/Safe Kids: Evaluation of a child sexual abuse prevention program.

Conte, J. R., Rosen, C., Saperstein, L., & Shermack, R. (1985). An evaluation of a program to prevent the sexual victimization of young children. *Child Abuse & Neglect*, *9*(3), 319-328.

Crowley, K. J. (1990). Evaluation of good touches/bad touches: A program to prevent child sexual abuse in school-age children.

Daigneault, I., Hébert, M., McDuff, P., & Frappier, J. Y. (2012). Evaluation of a sexual abuse prevention workshop in a multicultural, impoverished urban area. *Journal of Child Sexual Abuse*, *21*(5), 521-542.

Dake, J. A., Price, J. H., & Murnan, J. (2003). Evaluation of a child abuse prevention curriculum for third- grade students: assessment of knowledge and efficacy expectations. *Journal of School Health*, *73*(2), 76-82.

Dhooper, S. S., & Schneider, P. L. (1995). Evaluation of a school-based child abuse prevention program. *Research on Social Work Practice*, *5*(1), 36-46.

Gibson, L. E., & Leitenberg, H. (2000). Child sexual abuse prevention programs: Do they decrease the occurrence of child sexual abuse?. *Child Abuse & Neglect*, *24*(9), 1115-1125.

Fryda, C. M., & Hulme, P. A. (2015). School-based childhood sexual abuse prevention programs: An integrative review. *The Journal of School Nursing*, *31*(3), 167-182.

Fuqua, D. S. (2008). *Safe@ Last: The evaluation of a child sexual abuse prevention program for elementary students* (Doctoral dissertation, Tennessee State University).

Fryer Jr, G. E., Kraizer, S. K., & Mlyoshi, T. (1987). Measuring actual reduction of risk to child abuse: A new approach. *Child Abuse & Neglect*, *11*(2), 173-179.

Grendel, M. A. (1991). *Cognitive and emotional effects of a brief child sexual abuse prevention program for first graders* (Doctoral dissertation, University of Cincinnati).

Harvey, P., Forehand, R., Brown, C., & Holmes, T. (1988). The prevention of sexual abuse: Examination of the effectiveness of a program with kindergarten-age children. *Behavior Therapy*, *19*(3), 429-435.

Hazzard, A., Webb, C., Kleemeier, C., Angert, L., & Pohl, J. (1991). Child sexual abuse prevention: Evaluation and one-year follow-up. *Child Abuse & Neglect*, *15*(1-2), 123-138.

Hébert, M., Lavoie, F., Piché, C., & Poitras, M. (2001). Proximate effects of a child sexual abuse prevention program in elementary school children☆,☆☆. *Child abuse & neglect*, *25*(4), 505-522.

Herman, P. (1989). Evaluation of a sexual abuse curriculum/prevention program.

Ko, S. F. (2002). Evaluation of school-based child abuse prevention based on high school follow-up.

Kolko, D. J., Moser, J. T., & Hughes, J. (1989). Classroom training in sexual victimization awareness and prevention skills: An extension of the Red Flag/Green Flag people program. *Journal of Family Violence*, *4*(1), 25-45.

Kraizer, S. (1991). The Safe Child Program for the prevention of child abuse: Development and evaluation of a school-based curriculum.

Le Borgne, C., & Tisdall, E. K. M. (2017). Children's participation: Questioning competence and competencies?. *Social inclusion*, *5*(3), 122-130.

MacIntyre, D., & Carr, A. (1999). Evaluation of the effectiveness of the stay safe primary prevention programme for child sexual abuse. *Child Abuse & Neglect*, *23*(12), 1307-1325.

Morris, M. C., Kouros, C. D., Janecek, K., Freeman, R., Mielock, A., & Garber, J. (2017). Community-level moderators of a school-based childhood sexual assault prevention program. *Child abuse & neglect*, *63*, 295-306.

Oldfield, D., Hays, B. J., & Megel, M. E. (1996). Evaluation of the effectiveness of Project Trust: an elementary school-based victimization prevention strategy. *Child Abuse & Neglect*, *20*(9), 821-832.

Pawson, R., Greenhalgh, T., Harvey, G., & Walshe, K. (2004). Realist synthesis: an introduction. *Manchester: ESRC Research Methods Programme*, *University of Manchester*.

Poche, C., Yoder, P., & Miltenberger, R. (1988). Teaching self- protection to children using television techniques. *Journal of Applied Behavior Analysis*, *21*(3), 253-261.

Saslawsky, D. A., & Wurtele, S. K. (1986). Educating children about sexual abuse: implications for pediatric intervention and possible prevention1. *Journal of Pediatric Psychology*, *11*(2), 235-245.

Shearn, K., Allmark, P., Piercy, H., & Hirst, J. (2017). Building realist program theory for large complex and messy interventions. *International Journal of Qualitative Methods*, *16*(1), 1609406917741796.

Smothers, M. K., & Smothers, D. B. (2011). A sexual assault primary prevention model with diverse urban youth. *Journal of child sexual abuse*, *20*(6), 708-727.

Smylie, J., Kirst, M., McShane, K., Firestone, M., Wolfe, S., & O'Campo, P. (2016). Understanding the role of Indigenous community participation in Indigenous prenatal and infant-toddler health promotion programs in Canada: A realist review. *Social Science & Medicine*, *150*, 128-143.

Taal, M., & Edelaar, M. (1997). Positive and negative effects of a child sexual abuse prevention program. *Child abuse & neglect*, *21*(4), 399-410.

Telljohann, S. K., Everett, S. A., & Price, J. H. (1997). Evaluation of a third grade sexual abuse curriculum. *Journal of school health*, *67*(4), 149-153.

Volpe, R. (1984). A psychoeducational program dealing with child abuse for elementary school children. *Child abuse & neglect*, *8*(4), 511-517.

Warden, D., Moran, E., Gillies, J., Mayes, G., & Macleod, L. (1997). An evaluation of a children's safety training programme. *Educational Psychology*, *17*(4), 433-448.

Weatherley, R., Hajar, A. S., Noralina, O., John, M., Preusser, N., & Yong, M. (2012). Evaluation of a school-based sexual abuse prevention curriculum in Malaysia. *Children and Youth Services Review*, *34*(1), 119-125.

Wolfe, D. A., MacPherson, T., Blount, R., & Wolfe, V. V. (1986). Evaluation of a brief intervention for educating school children in awareness of physical and sexual abuse. *Child Abuse & Neglect*, *10*(1), 85-92.

Wurtele, S. K., Kast, L. C., & Melzer, A. M. (1992). Sexual abuse prevention education for young children: A comparison of teachers and parents as instructors. *Child Abuse & Neglect*, *16*(6), 865-876.

Yom, Y. H., & Eun, L. K. (2005). Effects of a CD-ROM educational program on sexual knowledge and attitude. *CIN: Computers, Informatics, Nursing*, *23*(4), 214-219.
